# Immunodominant and Neutralizing Linear B-Cell Epitopes Spanning the Spike and Membrane Proteins of Porcine Epidemic Diarrhea Virus

**DOI:** 10.3389/fimmu.2021.785293

**Published:** 2022-01-19

**Authors:** Kanokporn Polyiam, Marasri Ruengjitchatchawalya, Phenjun Mekvichitsaeng, Kampon Kaeoket, Tawatchai Hoonsuwan, Pichai Joiphaeng, Yaowaluck Maprang Roshorm

**Affiliations:** ^1^ Division of Biotechnology, School of Bioresources and Technology, King Mongkut’s University of Technology Thonburi, Bangkok, Thailand; ^2^ Bioinformatics and Systems Biology Program, School of Bioresources and Technology, King Mongkut’s University of Technology Thonburi, Bangkok, Thailand; ^3^ Pilot Plant Development and Training Institute, King Mongkut’s University of Technology Thonburi, Bangkok, Thailand; ^4^ Department of Clinical Sciences and Public Health, Faculty of Veterinary Sciences, Mahidol University, Salaya, Thailand; ^5^ B.F. Feed Company Limited, Bangkok, Thailand

**Keywords:** porcine epidemic diarrhea virus, spike protein, membrane protein, neutralizing epitope, immunoinformatics, epitope prediction, immunodominant epitope

## Abstract

Porcine epidemic diarrhea virus (PEDV) is the causative agent of PED, an enteric disease that causes high mortality rates in piglets. PEDV is an alphacoronavirus that has high genetic diversity. Insights into neutralizing B-cell epitopes of all genetically diverse PEDV strains are of importance, particularly for designing a vaccine that can provide broad protection against PEDV. In this work, we aimed to explore the landscape of linear B-cell epitopes on the spike (S) and membrane (M) proteins of global PEDV strains. All amino acid sequences of the PEDV S and M proteins were retrieved from the NCBI database and grouped. Immunoinformatics-based methods were next developed and used to identify putative linear B-cell epitopes from 14 and 5 consensus sequences generated from distinct groups of the S and M proteins, respectively. ELISA testing predicted peptides with PEDV-positive sera revealed nine novel immunodominant epitopes on the S protein. Importantly, seven of these novel immunodominant epitopes and other subdominant epitopes were demonstrated to be neutralizing epitopes by neutralization–inhibition assay. Our findings unveil important roles of the PEDV S2 subunit in both immune stimulation and virus neutralization. Additionally, our study shows the first time that the M protein is also the target of PEDV neutralization with seven neutralizing epitopes identified. Conservancy profiles of the epitopes are also provided. In this study, we offer immunoinformatics-based methods for linear B-cell epitope identification and a more complete profile of linear B-cell epitopes across the PEDV S and M proteins, which may contribute to the development of a greater next-generation PEDV vaccine as well as peptide-based immunoassays.

## Introduction

A swine disease named porcine epidemic diarrhea (PED) is one of the main diseases that causes huge economic losses in the swine industry worldwide, including Asia, America, and Europe (1, 2). Porcine epidemic diarrhea virus (PEDV) is the causative agent of PED (3). PEDV can infect and cause symptoms in all ages of pigs; however, it has more effect on suckling piglets with 80%–100% death rate (4). PEDV is a member of the *Alphacoronavirus* genus and it is an enveloped, positive-sense, single-stranded RNA virus (2). The PEDV genome is approximately 28 kb long that encodes three non-structural proteins and four structural proteins, which are spike protein (S), membrane protein (M), envelope protein (E), and nucleocapsid protein (N) (2).

Studies on the genetic profile of PEDV have demonstrated that the PEDV genome is highly diverse (5). Outbreaks and re-emergences of PEDV with high genetic diversity have been reported in many countries (6, 7). Even though vaccination is an effective method to prevent and control infection, the high genetic variation of PEDV remains one of the challenges in designing an effective PEDV vaccine. Although both B cells and T cells can be elicited by vaccines, it is generally thought that most vaccines confer protection through the induction of B cells to produce neutralizing antibodies (8). Hence, understanding of antibody responses following PEDV infection in regard to the landscape of immunodominant and neutralizing B-cell epitopes as well as conserved and unique epitopes in distinct strains will facilitate designing a more powerful universal vaccine that can cope with all diverse strains of PEDV.

While the M protein is the most abundant in the PEDV particle and plays an important role in the viral assembly process (9, 10), the PEDV S protein interacts with the host receptor and is composed of immunogenic regions capable of inducing neutralizing antibodies (11, 12). The S protein is thus considered the main target for vaccination. Similar to other coronaviruses, the PEDV S protein is a large glycoprotein composed of 1,383 amino acids (based on the classical strain PEDV CV777), and it can be divided into two functional subunits: i) the N-terminal S1 subunit, responsible for receptor binding, and ii) the C-terminal S2 subunit, responsible for membrane fusion (13, 14). The S1 subunit is comprised of the N-terminal domain (NTD) and the CO-26K equivalent (COE) domain (residues 499–638), which is responsible for receptor binding (13, 15). The S2 subunit consists of three domains: a large ectodomain, a transmembrane domain, and a cytoplasmic tail or endodomain (13, 16). A large ectodomain is composed of protease cleavage site, fusion peptide (FP), and two heptad repeat (HR1 and HR2) regions, which play important roles in viral and host cell membrane fusion (13, 16, 17).

The COE domain has been identified as an immunogenic domain containing B-cell epitopes recognized by neutralizing antibodies (12, 15, 18); therefore, it is considered an alternative vaccine target and has been extensively used in the development of recombinant PEDV vaccines (19–21). In the M protein, an epitope named M-14 has been identified from the PEDV CH/SHH/06 strain (9). In the S protein, four linear B-cell epitopes, namely, i) S1D5 with SS2 as a core epitope (22), ii) S1D6 with SS6 as a core epitope (22), iii) peptide M (23), and iv) 2C10, a neutralizing B-cell epitope located at the C-terminus of the S protein (24, 25), were first identified. More recently, two conformational neutralizing epitopes located in the S1 NTD and COE were identified from truncated S proteins of the PEDV PT strain (12). Additionally, neutralizing epitopes located at the same region with the S1D5 and S1D6 epitopes were reported (11, 22). All these epitopes were identified based on experimental methods such as ELISA with truncated proteins, pepscan, and phage display, which are laborious, costly, and time-consuming. Additionally, by using these techniques, B-cell epitope identification can focus only on some regions of the proteins, while a complete profile of B-cell epitope across the entire proteins is of importance for vaccine design and antibody detection.

Recently, immunoinformatics has been demonstrated to be a powerful tool for the identification of B- and T-cell epitopes (26, 27) as well as for vaccine design and *in-silico* evaluation (28, 29). Importantly, the immunoinformatics approach can facilitate big data analysis with less cost and time but high output compared with experimental methods. Currently, there are many tools available for *in-silico* B-cell epitope prediction. Among the epitope prediction resources, the IEDB database provides multiple tools for B-cell epitope prediction with high accuracy (30). Generally, the peptides predicted by immunoinformatics methods cannot be claimed as epitopes, although they are well characterized as potential B-cell epitopes. Thus, experimental validation is crucial and necessary for confirming whether predicted epitopes are genuine epitopes. Based on this combined method, a complete set of B-cell epitopes across the entire protein can be identified.

In the present work, we aimed to identify linear B-cell epitopes of two PEDV structural proteins, S and M, from all diverse strains available in the database and to characterize their potential as neutralizing epitope. The amino acid sequences of the PEDV S and M available in the National Center for Biotechnology Information (NCBI) database were retrieved and a phylogenetic tree was generated. Consensus sequences obtained from each group were then subject to linear B-cell epitope prediction using multiple immunoinformatics tools. We developed our own prediction methods for identifying potential B-cell epitopes based on four known epitopes [S1D5 (SS2), S1D6 (SS6), peptide M, and 2C10]. The predicted peptides were then experimentally validated using ELISA by testing synthetic peptides with PEDV-positive sera. Their potential as a neutralizing epitope was then investigated using neutralization–inhibition assay. Based on this approach, the whole set of neutralizing linear B-cell epitopes as well as immunodominant epitopes across the PEDV S and M protein was identified.

## Results

### The S and M Proteins From Global PEDV Strains Are Classified Into 14 and 5 Groups

**Figure 1:**
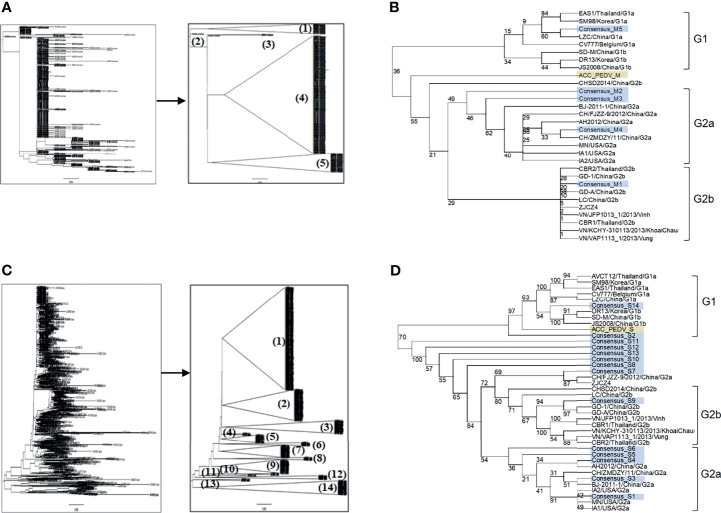
Grouping of the retrieved PEDV M and S proteins and phylogenetic tree analysis of the consensus sequences. **(A, C)** Phylogenetic tree and grouping of the retrieved PEDV M and S proteins, respectively. Phylogenetic trees were generated using the SeaView program (left panel) and groups were subsequently generated using the FigTree tool (right panel). **(B, D)** Phylogenetic tree analysis of the consensus sequences of the M **(B)** and S **(D)** proteins, respectively. The consensus sequences generated from each group of the proteins S and M were analyzed and grouped with PEDV from different genotypes using the SeaView program. The amino acid sequences were aligned using MUSCLE and trees were constructed based on distance analysis, NJ method, and bootstrap 1,000 replication. Numbers at nodes represent the percentage of 1,000 bootstrap replicates. ACC_PEDV (highlighted in yellow) is the virus used in the assays. Consensus sequences are highlighted in gray.

Amino acid sequences of the PEDV S and M proteins were retrieved from all sequences in the NCBI database. After filtering with Sublime Text 3 to remove incomplete and repeated sequences, 560 and 924 sequences of the M and S proteins were obtained, respectively. The sequences of each protein were next grouped and phylogenetic trees were generated. Sequences of the M protein were classified into 5 groups, while sequences of the S protein were classified into 14 groups (**Figures 1A, C** **)**. The consensus sequence of each group of both proteins was next generated and aligned as shown in **Supplementary Figures 1, 2**. Percent similarity among different groups was in the range from 32% to 99.71% for the S protein and from 20.35% to 99.55% for the M protein. The M and S consensus sequences were subsequently aligned with representative sequences of each genotype and subgenotype of PEDV and a phylogenetic tree was generated using the SeaView program. Consensus sequences of groups M1–M4 were clustered in genotype G2 and only consensus sequence of the M5 group was clustered in genotype G1 which is genetically related to CV777 (**Figure 1B** **)**. For the S protein, only the consensus sequences of groups S2 and S14 were clustered in genotype G1, while all the other sequences were clustered in genotype G2 (**Figure 1D**).

### Three Prediction Methods Are Developed and Used to Identify Linear B-Cell Epitopes

**Figure 2:**
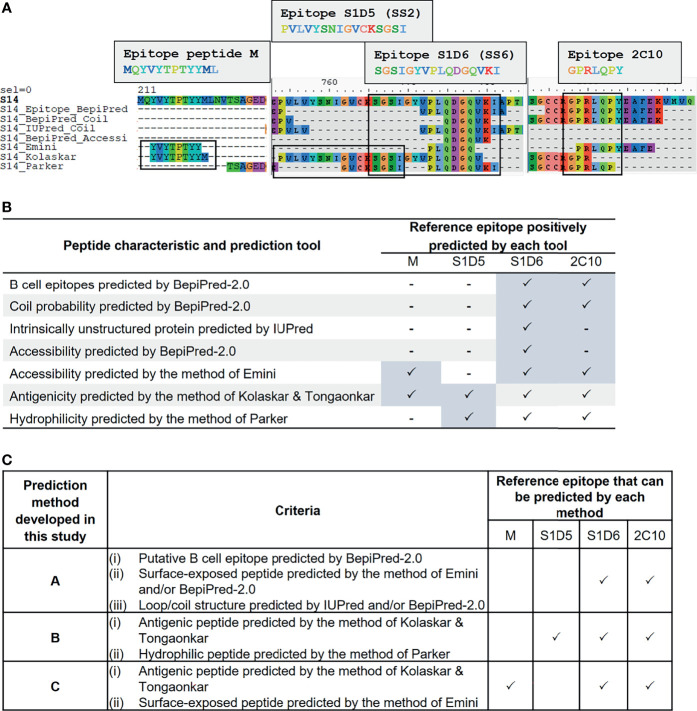
Development of immunoinformatics-based methods for linear B-cell prediction. Amino acid sequence of the S protein was predicted for various characteristics including probability as B-cell epitope, coil structure, surface accessibility, antigenicity, and hydrophilicity using multiple immunoinformatics prediction tools as indicated in **(A, B)**. Predicted peptides obtained from each tool were aligned and mapped to reference epitopes, which are four published epitopes: peptide M, S1D5, S1D6, and 2C10 **(A)**. Tools that gave positive prediction for the four reference epitopes are summarized in **(B)**. Three immunoinformatics-based prediction methods were subsequently developed and designated methods **(A–C)** with criteria indicated in **(C)**.

We employed immunoinformatics to screen for B-cell epitopes from diverse PEDV strains. Linear B-cell epitopes have been suggested to be correlated with surface accessibility, flexibility, coil probability, antigenicity, and hydrophilicity (31, 32); thus, we utilized multiple immunoinformatics tools to predict linear B-cell epitopes and peptide characteristics. BepiPred-2.0 was used to predict linear B-cell epitopes. In parallel, IUPred was used to predict intrinsically unstructured proteins inferring coil probability, while other features including accessibility, antigenicity, and hydrophilicity were predicted using the methods of Emini, Kolaskar and Tongaonkar, and Parker, respectively (**Figure 2A**).

To search for a prediction tool/method that gives the best performance in terms of accuracy and epitope coverage, we aligned and compared all predicted peptides obtained from each prediction tool with the reference epitopes. Four well-known B-cell epitopes of the PEDV S protein, namely, i) peptide M (MQYVYEPTYYML) (23), ii) S1D5 (PVLVYSNIGVCKSGSI) [with the core epitope SS2 (YSNIGVCK)] (22), iii) S1D6 (SGSIGYVPLQDGQVKI) [with the core epitope SS6 (LQDGQVKI)] (22), and iv) 2C10 (GPRLQPY) (24, 25), were exploited as reference epitopes (**Figure 2A**). Mapping of the reference epitopes with predicted peptides revealed that none of the prediction tools could positively predict all four reference epitopes (**Figures 2A, B** **)**; we, thus, created three new prediction methods, designated methods A, B, and C, for identifying and selecting linear B-cell epitopes as described in **Figure 2C**. In method A, epitope is identified based on the peptide with the following characteristics: i) predicted to be B-cell epitope by BepiPred, ii) composed of coil structure predicted by BepiPred and/or IUPred, and iii) accessible peptide predicted by BepiPred and/or Emini method. For method B, the epitopes are identified based on the peptides predicted to be antigenic and hydrophilic by the methods of Kolaskar and Tongaonkar and Parker, respectively. For method C, the peptides predicted to be antigenic and accessible by the methods of Kolaskar and Tongaonkar and Emini, respectively, are considered B-cell epitope. By using our prediction methods, the reference epitopes S1D6 and 2C10 were positively predicted by all three methods, while epitope S1D5 and peptide M were positively predicted only by methods B and C, respectively.

### Potential Linear B-Cell Epitopes Are Identified in 7 and 33 Regions of the PEDV M and S Proteins

**Figure 3:**
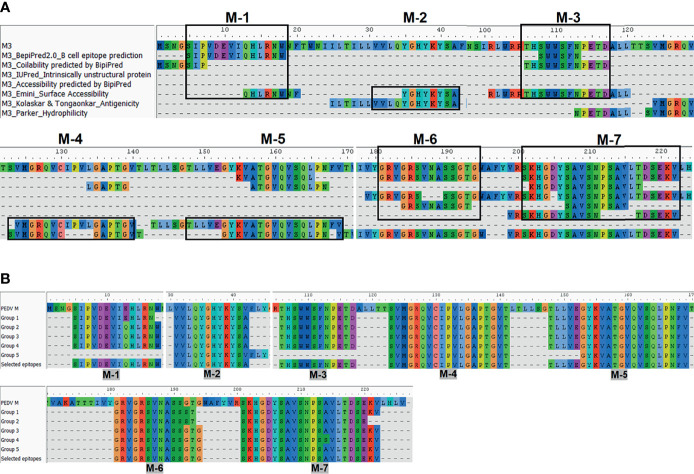
Immunoinformatics prediction of the linear B-cell epitopes from the consensus sequence of the M protein. Consensus sequences from the M protein were subject to B-cell epitope prediction using prediction methods describe in **Figure 2A** **(A)**. Consensus sequence from group M-3 is shown as a representative. Linear B-cell epitopes were then identified based on the criteria of those three prediction methods. Predicted peptides obtained from all five groups of the M protein were then aligned **(B)**. Peptides selected for further experimental validation are indicated in the “selected epitope” line with the designated name.

**Figure 4:**
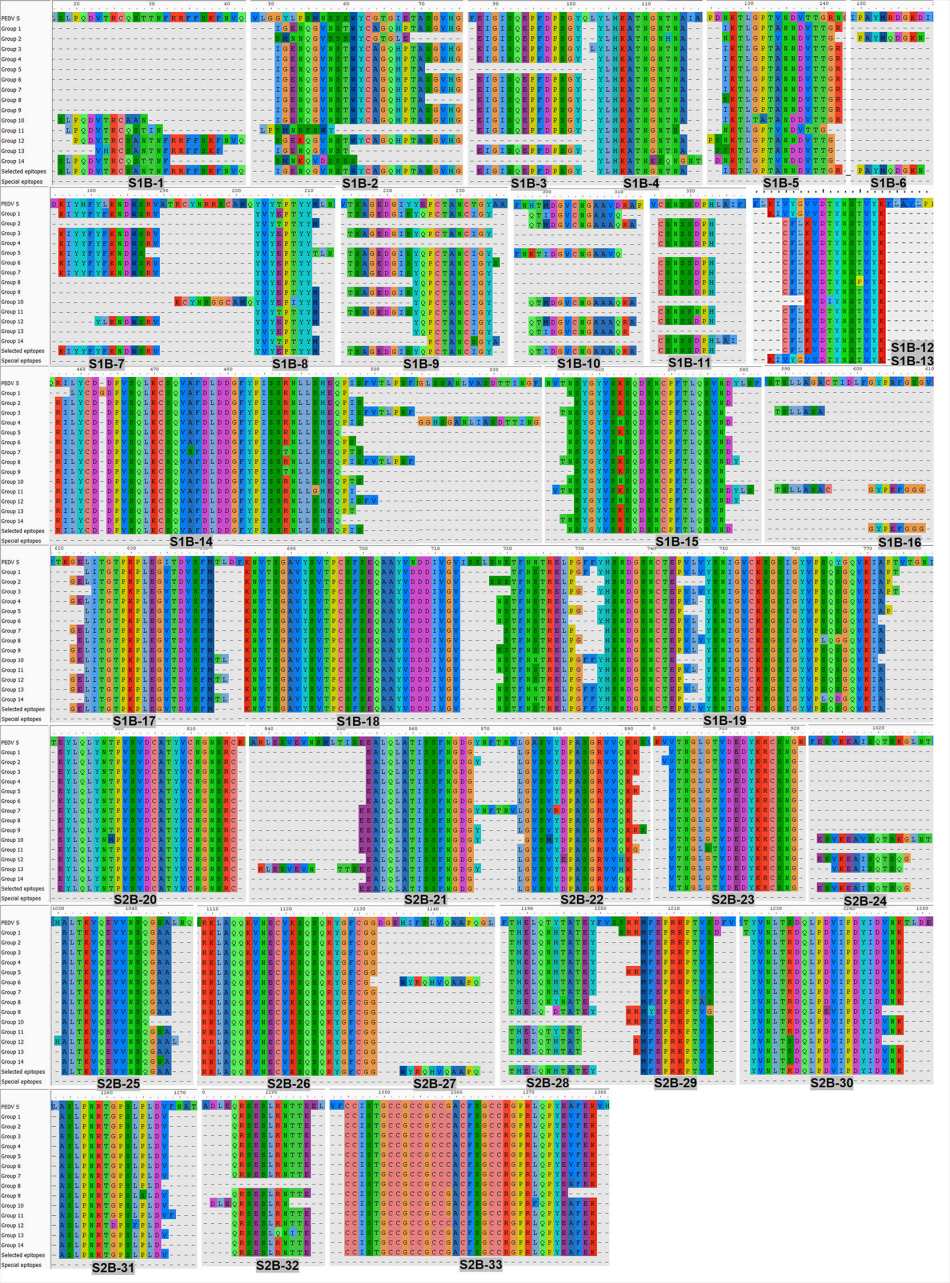
Predicted B-cell epitopes of the PEDV S protein. B-cell epitopes were predicted from the consensus sequences of 14 groups of the S protein. Linear B-cell epitopes were identified following the three prediction methods described in **Figure 2**. Peptides resulting from the prediction of all 14 groups of the S protein were aligned and the peptides selected for further experimental evaluation are indicated in the “selected epitope” line with the designated name.

The consensus sequences from all 14 groups of the S protein and 5 groups of the M protein were subject to linear B-cell epitope prediction. Putative B-cell epitopes were identified and selected based on prediction methods A, B, and C, and prediction of the M protein group 3 (M3) is shown as a representative (**Figure 3A**). Note that the predicted peptides that overlap or are located in close proximity were combined into one long peptide. Prediction of the M protein yielded seven putative B-cell epitopes, designated M-1 to M-7 (**Figures 3A, B** and **Table 1**). Prediction of B-cell epitopes on the S protein from one representative group (out of 14 groups) using our methods is shown in **Supplementary Figure 3**. Predicted peptides from 14 S consensus sequences were aligned, and a total of 50 peptides located in 33 regions of the S protein, designated S1B and S2B for the epitopes located on the S1 and S2 subunits, respectively, were predicted as B-cell epitopes (**Figure 4**, **Table 1**). In the COE region, three epitopes, namely, S1B-15, S1B-16, and S1B-17, were identified. Most of the B-cell epitopes predicted from consensus sequences of different groups are located on the same regions although some residues in the epitope are variable among different groups. Impressively, our immunoinformatics methods could identify all published epitopes in the S protein but not in the M protein as indicated in **Table 1** and **Supplementary Figures 4, 5**.

**Table 1:** Predicted B-cell epitopes of the PEDV M and S proteins that were selected for further experimental validation.

Peptide Name	Position^*^ (Start–End)	Corresponding Group	Predicted Peptide	Length	Method	Published Epitope
M-1	5–18	M 2–5	SIPVDEVIQHLRNW	14	A	
M-2	31–42	M 1–5	VVLQYGHYKYSA	12	C	
M-3	106–117	M 1–5	THSWWSFNPETD	12	A	
M-4	123–141	M 1–5	SVMGRQVCIPVLGAPTGVT	19	B	
M-5	148–169	M 1–5	TLLVEGYKVATGVQVSQLPNFV	22	B	
M-6	181–194	M 3–5	GRVGRSVNASSGTG	14	A	
M-7	201–222	M 1–3, 5	SKHGDYSAVSNPSAVLTDSEKV	22	A	
S1B-1	18–42	S 1–9, 11–13	SLPQDVTRCSANTNFRRFFSKFNVQ	25	A	
S1B-2	51–75	S 1–13	IGENQGVNSTWYCAGQHPTASGVHG	25	Combined	
S1B-2.1	51–70	S 1–13	IGENQGVNSTWYCAGQHPTA	20	A	
S1B-2.2	65–75	S 1–14	GQHPTASGVHG	11	B	
S1B-3	88–101	S 1–14	EIGISQEPFDPSGY	14	A	
S1B-4	104–115	S 1–14	YLHKATNGNTNA	12	A	
S1B-5	127–142	S 1–9, 11, 12	IKTLGPTANNDVTTGR	16	A	
S1B-6	150–158	S 2	PAYMQDGKN	9	A	
S1B-7	176–189	S 1–9, 12–14	KIYYFYFKNDWSRV	14	A	
S1B-8	203–211	S 1–9, 11–14	YVYEPTYYM	9	C	Peptide M (23)
S1B-9	215–234	S 1–14	TSAGEDGISYQPCTANCIGY	20	Combined	
S1B-9.1	215–226	S 1–14	TSAGEDGISYQP	12	A	
S1B-9.2	223–234	S 1–14	SYQPCTANCIGY	12	B	
S1B-10	298–312	S 2, 7, 13	QTIDGVCNGAAAQRA	15	A	
S1B-11	346–353	S 1–9, 12, 14	CSNSSDPH	8	B	
S1B-12	370–383	S 1–9, 11–14	CFLKVDTYNSTVYK	14	B, C	
S1B-13	368–383	S 1	KIVYGVVDTYNSTVYK	16	A, B, C	
S1B-14	457–497	S 1–4, 6, 8, 9, 11, 12, 14	RILYCDDPVSQLKCSQVAFDLDDGFYPISSRNLLSHEQPIS	41	Combined	aa. 435–484 (12)
S1B-14.1	457–477	S 1–14	RILYCDDPVSQLKCSQVAFDL	21	B	
S1B-14.2	475–497	S 1–4, 6–9, 11, 12, 14	FDLDDGFYPISSRNLLSHEQPIS	23	A	
S1B-15	556–578	S 1–3, 5, 7–9, 11–14	NSYGYVSKSQDSNCPFTLQSVND	23	A	aa. 575–639 (12)
S1B-16	602–609	S 1–3, 7, 11	GYPEFGGG	8	A	C2-1 (18), aa. 575–639 (12)
S1B-17	622–641	S 1–14	GELITGTPKPLEGVTDVSFM	20	A	aa. 575–639 (12)
S1B-18	684–713	S 1–14	KNVTSGAVYSVTPCSFSEQAAYVDDDIVGV	30	B	
S1B-19	719–772	S 1–9, 11, 12	NSTFNSTRELPGFFYHSNDGSNCTEPVLVYSNIGVCKSGSIGYVPSQSGQVKIA	54	Combined	744–774 (11)
S1B-19.1	719–730	S 1–9, 11, 12	NSTFNSTRELPG	12	A	SE16 (33)
S1B-19.2	733–746	S 1–14	YHSNDGSNCTEPVL	14	A	
S1B-19.3	748–772	S 1–9, 11–14	YSNIGVCKSGSIGYVPSQSGQVKIA	25	A, B, C	SS2 and SS6 (22)
S2B-20	792–816	S 1–9, 11–14	EYLQLYNTPVSVDCATYVCNGNSRC	25	Combined	
S2B-20.1	792–805	S 1–9, 11–14	EYLQLYNTPVSVDC	14	C	
S2B-20.2	795–816	S 1–9, 11–14	QLYNTPVSVDCATYVCNGNSRC	22	B	
S2B-21	853–868	S 1–14	EEALQLATISSFNGDG	16	A	
S2B-22	875–890	S 1–11, 14	LGVSVYDPASGRVVQK	16	A, B	
S2B-23	903–920	S 1–10, 12–14	VTNGLGTVDEDYKRCSNG	18	A	
S2B-24	1,012–1,024	S 12–14	ESVKEAISQTSQG	13	A	
S2B-25	1,031–1,045	S 1–9, 12–14	ALTKVQEVVNSQGAA	15	B	
S2B-26	1,109–1,132	S 1–11, 13, 14	RKLAQQKVNECVKSQSQRYGFCGG	24	Combined	
S2B-26.1	1,109–1,121	S 1–11, 13, 14	RKLAQQKVNECVK	13	B	
S2B-26.2	1,119–1,132	S 1–14	CVKSQSQRYGFCGG	14	A	
S2B-27	1,136–1,146	–	WYRQHVQAAPQ	11	A, B, C	
S2B-28	1,188–1,199	S 1–7, 14	THELQNHTATEY	12	A	
S2B-29	1,206–1,215	S 1–7, 10–14	MFEPRKPTVS	10	A	
S2B-30	1,227–1,247	S 1–11	YVNLTRDQLPDVIPDYIDVNK	21	Combined	
S2B-30.1	1,227–1,235	S 1–11	YVNLTRDQL	9	B, C	
S2B-30.2	1,230–1,247	S 1–11	LTRDQLPDVIPDYIDVNK	18	A	
S2B-31	1,254–1,268	S 1–8, 10, 11, 13, 14	ASLPNRTGPSLPLDV	15	A	
S2B-32	1,285–1,295	S 1–7, 9, 11, 12, 14	QRSESLRNTTE	11	A	
S2B-33	1,345–1,379	S 2, 7–9, 11, 13, 14	CCISTGCCGCCGCCGACFSGCCRGPRLQPYEAFEK	35	Combined	
S2B-33.1	1,345–1,373	S 2, 7–9, 11, 13, 14	CCISTGCCGCCGCCGACFSGCCRGPRLQP	29	B	
S2B-33.2	1,365–1,379	S 1–3, 6–9, 11–14	CCRGPRLQPYEAFEK	15	A	2C10 (24)

* Positions of amino acid residues are based on the M and S amino acid sequences of PEDV CV777.

### B-Cell Epitopes on the S2 Subunit and M Protein Are More Conserved Than Those on the S1 Subunit

**Figure 5:**
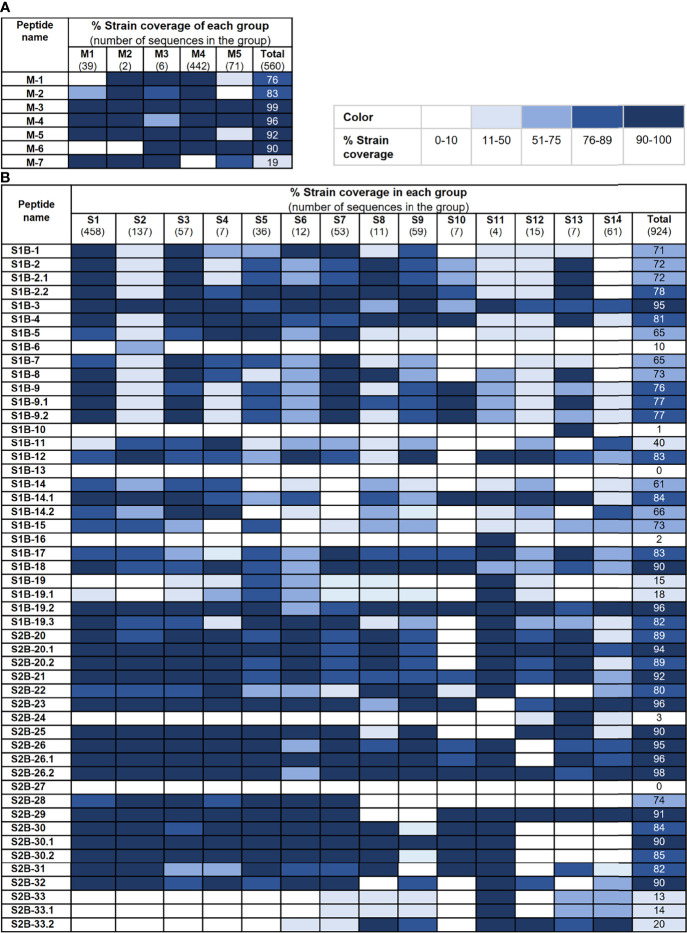
Conservancy of each predicted epitope among global PEDV strains. Strain coverage of the predicted epitopes of the M and S proteins are shown in **(A, B)**, respectively. Percentage of strain coverage was calculated based on 100% amino acid sequence identity between the predicted epitope and the target sequence.

We further analyzed the strain coverage of each predicted epitope as information toward epitope conservancy could be beneficial to the design and development of a vaccine, particularly a universal vaccine. For the M protein, six out of seven predicted epitopes cover higher than 70% of 560 accession numbers with four epitopes (M-3, M-4, M-5, and M-6) having strain coverage greater than 90% (**Figure 5A**). Analyzed with 924 accession numbers of the PEDV S protein, 34 out of 50 predicted epitopes of the S protein exhibited strain coverage greater than 70% (**Figure 5B**). Among these 34 epitopes, 24 showed strain coverage higher than 80%, of which 8 and 16 are located on the S1 and S2 subunits, respectively, suggesting that sequences in the S1 subunit are more variable than those in the S2 subunit.

### Predicted B-Cell Epitopes Are Recognized by PEDV-Positive Sera

**Figure 6:**
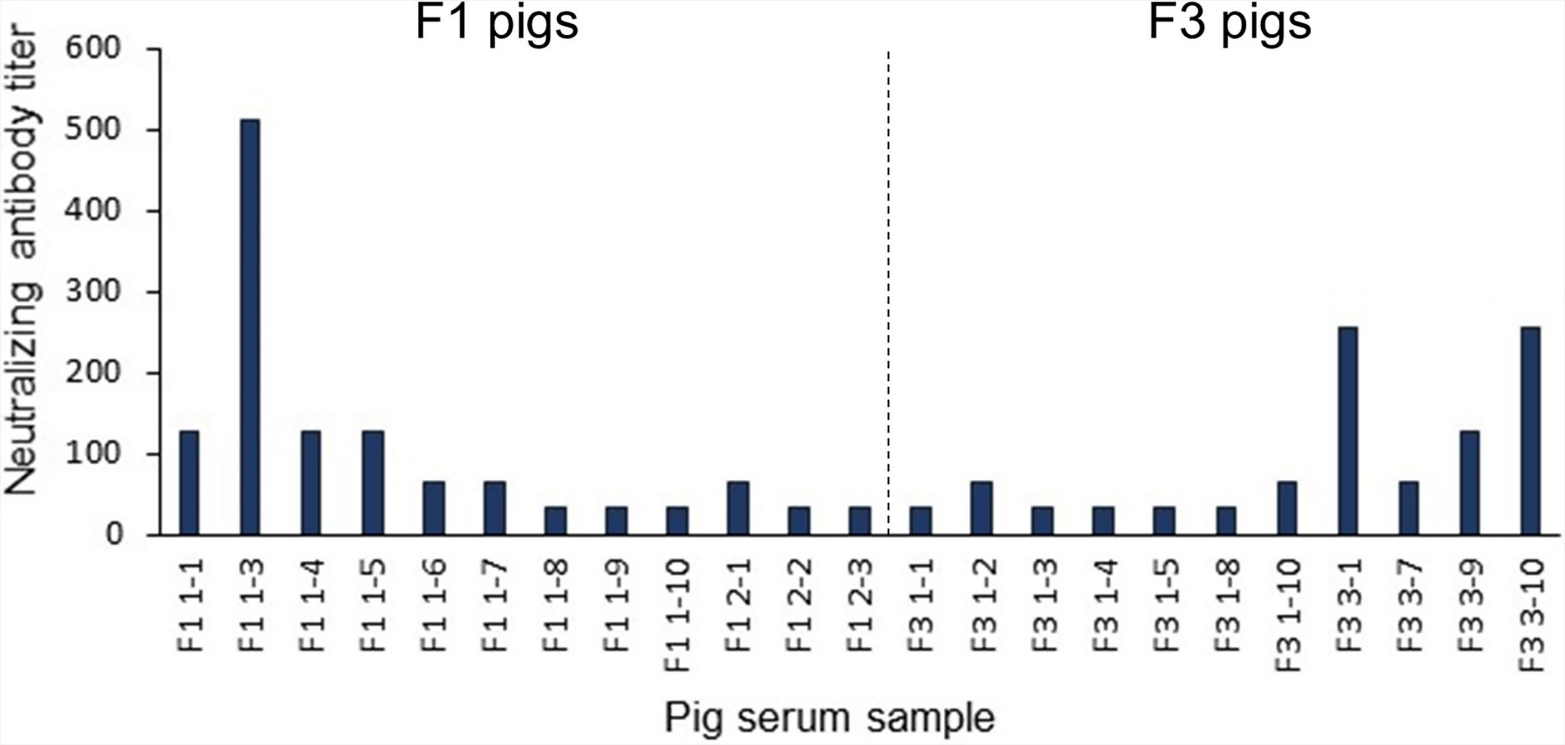
Neutralization titer of the pig sera. Pig sera were 2-fold serially diluted ranging from 1:32 to 1:4,096 and incubated for 1 h with PEDV (10 TCID_50_) before adding to Vero cells grown in 96-well plates (10 wells/dilution). PEDV infection was investigated by immunofluorescence staining using anti-PEDV monoclonal antibody at day 3 post-inoculation. Only serum samples with neutralization titer at least 32 are shown and chosen for further studies.

To further validate the predicted linear B-cell epitopes, ELISA was performed. PEDV-positive sera were prepared from F1 and F3 pigs raised in the farms and naturally infected with PEDV. Neutralizing activity of the pig sera was investigated using neutralization assay and only serum samples with neutralizing antibody titers at least 32 (reciprocal serum dilution) were further used in ELISA analysis. A total of 12 F1 pigs and 11 F3 pigs showed neutralizing activity that passes the criteria (**Figure 6**) and were subject to ELISA. Control sera were prepared from 10 uninfected F3 pigs raised in the animal research facility, and their neutralizing antibody titer was confirmed to be lower than 32 by neutralization assay (**Supplementary Table 1**).

**Figure 7:**
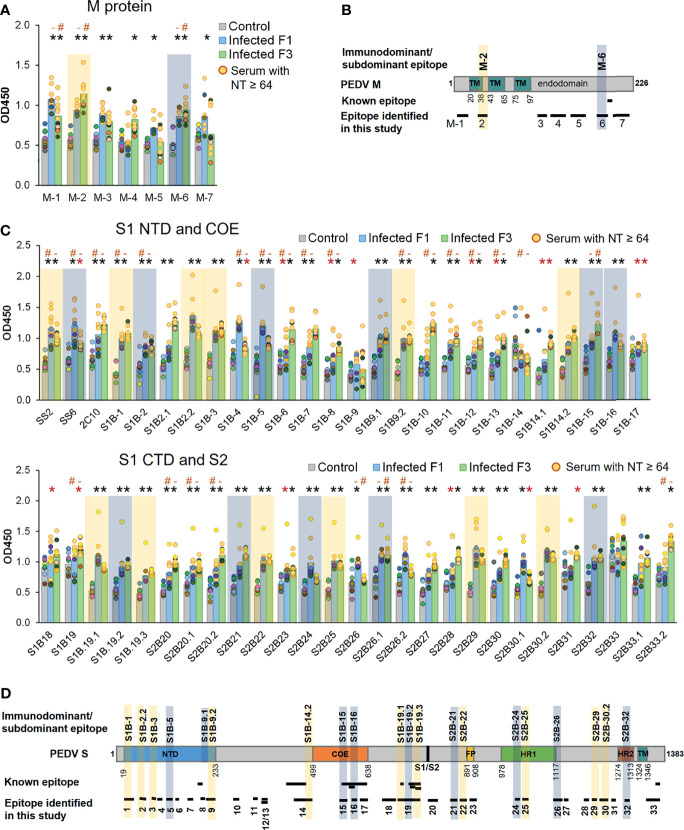
Antibody responses against predicted epitopes and landscapes of the immunodominant B-cell epitopes. **(A, C)** Antibody responses against predicted B-cell epitopes of the M and S proteins, respectively. ELISA was performed with PEDV-positive F1 (12 samples) and F3 (11 samples) pig sera whose neutralizing activity against PEDV had been confirmed. Sera from uninfected F3 pigs were used as a control. ELISA plates were coated with synthetic peptides corresponding to the predicted epitopes as indicated in the *X*-axis. Serum was diluted 1:50 and used in ELISA. The optical density at the wavelength of 450 nm was measured. Antibody response of the serum samples with neutralizing antibody titer (NT) ≥64 is shown in yellow circle. Non-parametric Mann–Whitney *U* test was used in statistical analysis. The significant difference of the infected F1 group vs control group and infected F3 group vs control group is indicated with * (red asterisk) indicates *p <*0.05 but >0.001; * (black asterisk) indicates *p <*0.001, ^#^ indicates a significant difference between sera with NT ≥64 and NT = 32 in reactivity with the same peptide. Immunodominant and subdominant epitopes were defined as follows. In reactivity with a particular peptide, if i) all sera from both F1 and F3 pigs exhibited OD450 value higher than OD450 mean of the control group + 2SD and ii) all sera from both F1 and F3 pigs exhibited OD450 value higher than OD450 mean of the control group + 1SD, such peptide was considered an i) immunodominant and ii) subdominant epitope, respectively. Immunodominant and subdominant are highlighted with yellow and blue box, respectively. **(B, D)** Landscape of the immunodominant B-cell epitopes on the PEDV M and S proteins, respectively. Positions of amino acid residues in the M and S proteins are based on the sequence of PEDV CV777 (accession no. AAK38659.1). The N-terminal domain (NTD), fusion peptide (FP), heptad repeat regions (HR1 and HR2), and transmembrane domain (TM) are indicated in the diagram. All immunodominant epitopes and some subdominant epitopes with their locations are indicated on the protein.

Three well known epitopes, SS2, SS6, and 2C10 (22, 25), were included in ELISA as reference epitopes. Reactivity of the peptides with their target antibodies was determined based on statistical analysis comparing OD450 value of the F1 and F3 pig sera to that of the control group (*p* < 0.05). All seven peptides from the M protein showed reactivity with sera from at least one pig strain (**Figure 7A**). Out of 50, 48 peptides of the S protein showed reactivity with the sera from at least one pig strain (**Figure 7C**). Immunodominant and subdominant epitopes were next defined. In reactivity with a particular peptide, if all sera from both F1 and F3 pigs exhibited OD450 value higher than OD450 mean of the control group + 2SD, such peptide was considered an immunodominant epitope. On the other hand, if all sera from both F1 and F3 pigs exhibited OD450 value higher than OD450 mean of the control group + 1SD, such peptide was considered a subdominant epitope. Based on these analyses, the epitopes S1B-1, S1B-2.2, S1B-3, S1B-9.2, S1B-14.2, S1B-19.1, S1B-19.3, S2B-22, S2B-25, S2B-29, and S2B-30.2 on the S protein and the epitope M-2 on the M protein were categorized as immunodominant epitopes, while the epitopes S1B-2, S1B-5, S1B-9.1, S1B-15, S1B-16, S1B-19.2, S2B-21, S2B-24, S2B-26.1, and S2B-32 were categorized as subdominant epitopes (**Figures 7A–D** and **Table 2** **)**. Two subdominant epitopes, S1B-15 and S1B-16, are located within the COE domain. Furthermore, we demonstrated that antibody response in the sera with high neutralizing titer (NT ≥ 64) and low neutralizing titer (NT = 32) against some epitopes was significantly different (*p* < 0.05) as indicated in **Figures 7A** **, C**. This information may suggest the correlation between antibodies targeting these epitopes and neutralizing activity of the serum.

**Table 2:** Potential as a neutralizing epitope determined by neutralizion-inhibition assay.

Peptide Name	Number of Sera That Have OD450 > OD450 Mean of the Control + 2SD		Epitope Dominance	Capability to Inhibit the Neutralizing Activity of the Serum			
	F1 pig (*n* = 12)	F3 pig (*n* = 11)		F1 1-3	F1 1-4	F1 1-5	F3 1-10
M-1	9/9 (*n* = 9)	6/11		nt	**✓**	**✓**	nt
M-2	9/9 (*n* = 9)	11/11	Immunodominant	nt	**✓**	**✓**	nt
M-3	9/9 (*n* = 9)	8/11		nt	**✓**	**–**	nt
M-4	2/9 (*n* = 9)	7/11		nt	**✓**	**–**	nt
M-5	9/9 (*n* = 9)	4/11		nt	**–**	**✓**	nt
M-6	9/9 (*n* = 9)	9/11	Subdominant	nt	**✓**	**✓**	nt
M-7	5/9 (*n* = 9)	3/11		nt	**–**	**✓**	nt
S1B-1	12/12	11/11	Immunodominant	nt	**✓**	**✓**	nt
S1B-2	8/12	6/11	Subdominant	**✓**	**✓**	**–**	**–**
S1B-2.1	9/12	11/11		**✓**	**–**	**–**	**–**
S1B-2.2	12/12	11/11	Immunodominant	**✓**	**✓**	**✓**	**✓**
S1B-3	12/12	11/11	Immunodominant	**✓**	**✓**	**–**	**–**
S1B-4	12/12	1/11		**✓**	**✓**	**–**	**✓**
S1B-5	12/12	2/11	Subdominant	nt	**✓**	**✓**	nt
S1B-6	4/12	11/11		**–**	**✓**	**–**	**–**
S1B-7	7/12	11/11		**–**	**–**	**–**	**–**
S1B-8	5/12	11/11		**✓**	**✓**	**–**	**✓**
S1B-9	7/12	4/11		**✓**	**✓**	**–**	**–**
S1B-9.1	11/12	11/11	Subdominant	**✓**	**–**	**–**	**✓**
S1B-9.2	12/12	11/11	Immunodominant	**✓**	**✓**	**–**	**✓**
S1B-10	5/12	11/11		**✓**	**✓**	**–**	**–**
S1B-11	10/12	11/11		**✓**	**–**	**–**	**–**
S1B-12	6/12	11/11		**–**	**✓**	**✓**	**✓**
S1B-13	4/12	11/11		**–**	**✓**	**✓**	**–**
S1B-14	0/12	0/11		**✓**	**–**	**–**	**–**
S1B-14.1	1/12	2/11		nt	**✓**	**–**	nt
S1B-14.2	12/12	11/11	Immunodominant	nt	**–**	**–**	nt
S1B-15	5/12	11/11	Subdominant	**✓**	**–**	**–**	**–**
S1B-16	12/12	9/11	Subdominant	**✓**	**✓**	**–**	**✓**
S1B-17	1/12	1/11		**✓**	**✓**	**–**	**✓**
S1B-18	4/12	2/11		**–**	**–**	**–**	**–**
S1B-19	1/12	3/11		**✓**	**–**	**–**	**–**
S1B-19.1	12/12	11/11	Immunodominant	nt	**–**	**–**	nt
S1B-19.2	11/12	11/11	Subdominant	nt	**–**	**–**	nt
S1B-19.3	12/12	11/11	Immunodominant	nt	**✓**	**–**	nt
S2B-20	8/12	11/11		**–**	**✓**	**✓**	**–**
S2B-20.1	8/12	8/11		**–**	**–**	**✓**	**✓**
S2B-20.2	7/12	11/11		**–**	**✓**	**–**	**✓**
S2B-21	9/12	11/11	Subdominant	**✓**	**–**	**–**	**✓**
S2B-22	12/12	11/11	Immunodominant	nt	**–**	**–**	nt
S2B-23	5/12	4/11		nt	**✓**	**–**	nt
S2B-24	12/12	3/11	Subdominant	nt	**✓**	**–**	nt
S2B-25	12/12	11/11	Immunodominant	nt	**✓**	**–**	nt
S2B-26	3/12	0/11		**✓**	**–**	**✓**	**✓**
S2B-26.1	6/12	5/11	Subdominant	**✓**	**✓**	**✓**	**✓**
S2B-26.2	10/12	1/11		**✓**	**–**	**✓**	**–**
S2B-27	7/12	11/11		**✓**	**✓**	**✓**	**✓**
S2B-28	5/12	11/11		**–**	**✓**	**✓**	**✓**
S2B-29	12/12	11/11	Immunodominant	**✓**	**✓**	**✓**	**✓**
S2B-30	2/12	11/11		**✓**	**✓**	**✓**	**✓**
S2B-30.1	12/12	2/11		**–**	**✓**	**–**	**–**
S2B-30.2	12/12	11/11	Immunodominant	**✓**	**✓**	**–**	**✓**
S2B-31	1/12	1/11		**✓**	**✓**	**–**	**✓**
S2B-32	4/12	11/11	Subdominant	**✓**	**–**	**–**	**–**
S2B-33	0/12	4/11		**✓**	**✓**	**✓**	**✓**
S2B-33.1	8/12	11/11		**✓**	**✓**	**✓**	**✓**
S2B-33.2	5/12	11/11		**–**	**✓**	**–**	**✓**

nt, not tested.

Based on the B-cell epitopes we identified in the present study, some have already been reported. The epitope S1B-19 (aa. 719–772) is a part of the S1D domain (aa. 636–789) containing neutralizing epitopes (34). The S1B-19.1 epitope (aa. 719–730) overlaps with the known epitope S1D SE16 (722-SSTFNSTREL-731) (33), while the S1B-19.3 epitope (aa. 748–772) consists of two known epitopes, SS2 (22) and SS6 (22). The S1B-8 epitope (aa. 203–211) corresponds to the known epitope, peptide M (23), while the epitope S2B-33 (aa. 1,345–1,379) contains the 2C10 epitope (25). The epitope S1B-14.1 (aa. 457–477) entirely maps in the neutralizing epitope (aa. 432–481) (12). In the COE region, all three epitopes (S1B-15 to S1B-17) are parts of the conformational epitope (572-TLQSVNDYLSFSKFCVSTSLLASACTIDLFGYPEFGSGVKFTSLYFQFTKGELITGTPKPLEGVT-636) (12) as underlined. Additionally, the S1B-16 epitope also has three amino acids (GYP) overlapping with the C2-1 epitope (TSLLASACTIDLFGYP) (18). Besides these known epitopes, other B-cell epitopes identified in our study are considered novel. Thus, epitopes S1B-1, S1B-2.2, S1B-3, S1B-9.2, S1B-14.2, S2B-22, S2B-25, S2B-29, and S2B-30.2 can be recognized as novel immunodominant epitopes in the PEDV S protein. For the M protein, one B-cell epitope named M14 (9) has been reported; however, this epitope does not overlap with the epitopes identified in our study. Therefore, all seven B-cell epitopes in the M protein we identified are novel epitopes.

### Novel Neutralizing Epitopes Are Verified by Neutralization–Inhibition Assay

**Figure 8:**
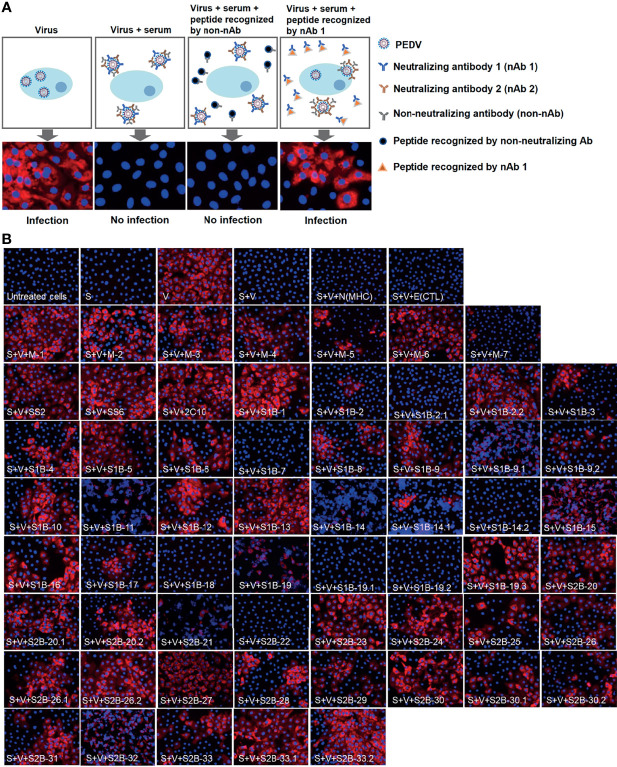
Neutralization–inhibition assay. **(A)** Schematic representation of neutralization–inhibition assay. Inhibitory effect of a peptide on the neutralizing activity of the serum was investigated at endpoint neutralization (with serum diluted to endpoint neutralization titer). **(B)** Inhibition of PEDV neutralization by peptides corresponding to B-cell epitopes. Peptides (as indicated in the picture) were incubated with sera from four PEDV-infected F1 (F1 1-3, 1-4, 1–5) and F3 1-10 pigs, prior to a further incubation with PEDV. The mixture was inoculated into Vero cells and incubated for 3 or 5 days. PEDV was detected using anti-PEDV monoclonal antibody and shown by red fluorescence. DAPI was used to determine the nucleus (blue fluorescence). Immunofluorescence images were observed and taken under an inverted fluorescence microscope. S, serum; V, virus. N(MHC) and E(CTL) are irrelevant peptides.

Neutralization ability of the antibodies against each epitope were further tested using neutralization–inhibition assay as shown in the schematic representation in **Figure 8A**. The assay was conducted with serially diluted serum and 100 TCID_50_ of PEDV. Neutralization inhibition mediated by a peptide was determined at the endpoint neutralizing antibody titer, at which the amount of antibodies is only sufficient to neutralize the virus and depletion of neutralizing antibodies will result in the loss of the neutralization potential of the serum. Four sera including F1 1-3, F1 1-4, F1 1-5, and F3 1-10 were used in the assay. Peptides corresponding to the known epitopes SS2, SS6, and 2C10 were used as references, and irrelevant peptides N(MHC) and E(CTL) were included as negative controls. All three reference peptides exhibited their ability to inhibit the neutralizing activity of the sera, while irrelevant peptides did not (**Figure 8B**). Notably, peptides from the M protein were tested with only two sera, F1 1-4 and F1 1-5. While the F1 1-4 serum lost its neutralizing activity when incubated with peptides M-1, M-2, M-3, M-4, and M-6, the neutralizing activity of serum F1 1-5 was depleted in the presence of peptides M-1, M-2, M-5, M-6, and M-7 (**Figure 8B**, **Supplementary Figures 7, 8** **)**. A combined result from both sera suggested that all seven epitopes of the M protein had the potential to be neutralizing epitopes. However, the three M peptides, namely, M-1, M-2, and M-6, could inhibit the neutralizing activity of both sera tested, suggesting them to be the most promising targets in the M protein for virus neutralization.

In the presence of the S peptides, four pig sera showed different patterns of neutralization inhibition as summarized in **Table 2**, **Supplementary Figures 6–9**. In **Figure 8B**, neutralizing epitopes were concluded based on the combined results of all four sera. The peptide that inhibited the neutralizing activity of at least one serum was considered a neutralizing epitope. Almost all peptides, except S1B-7, S1B-11, S1B-14.2, S1B-19.1, S1B-19.2, and S2B-22, could inhibit the neutralizing activity of at least one serum. Hence, epitopes S1B-1, S1B-2.2, S1B-3, S1B-9.2, S1B-19.3, S2B-25, S2B-29, and S2B-30.2 posed as both neutralizing and immunodominant epitopes. Importantly, antibodies recognizing the three epitopes in the COE domain, which are S1B-15, S1B-16, and S1B-17, were demonstrated with neutralization ability. Among the peptides capable of inhibiting the neutralizing activity of the serum, peptides S1B1, S1B-2.2, S1B-5, S2B-26.1, S2B-27, S2B-29, S2B-30, and S2B-33 showed neutralization inhibition in all sera tested, strongly confirming them as promising targets for PEDV neutralization. While most of the former epitope identifications focus on the S1 subunit, our work demonstrates that the S2 subunit indeed harbors multiple immunodominant as well as neutralizing epitopes, representing targets for both vaccine development and antibody detection.

### Depiction of the Identified B-Cell Epitopes in the 3-D Structure of the PEDV S Protein

**Figure 9:**
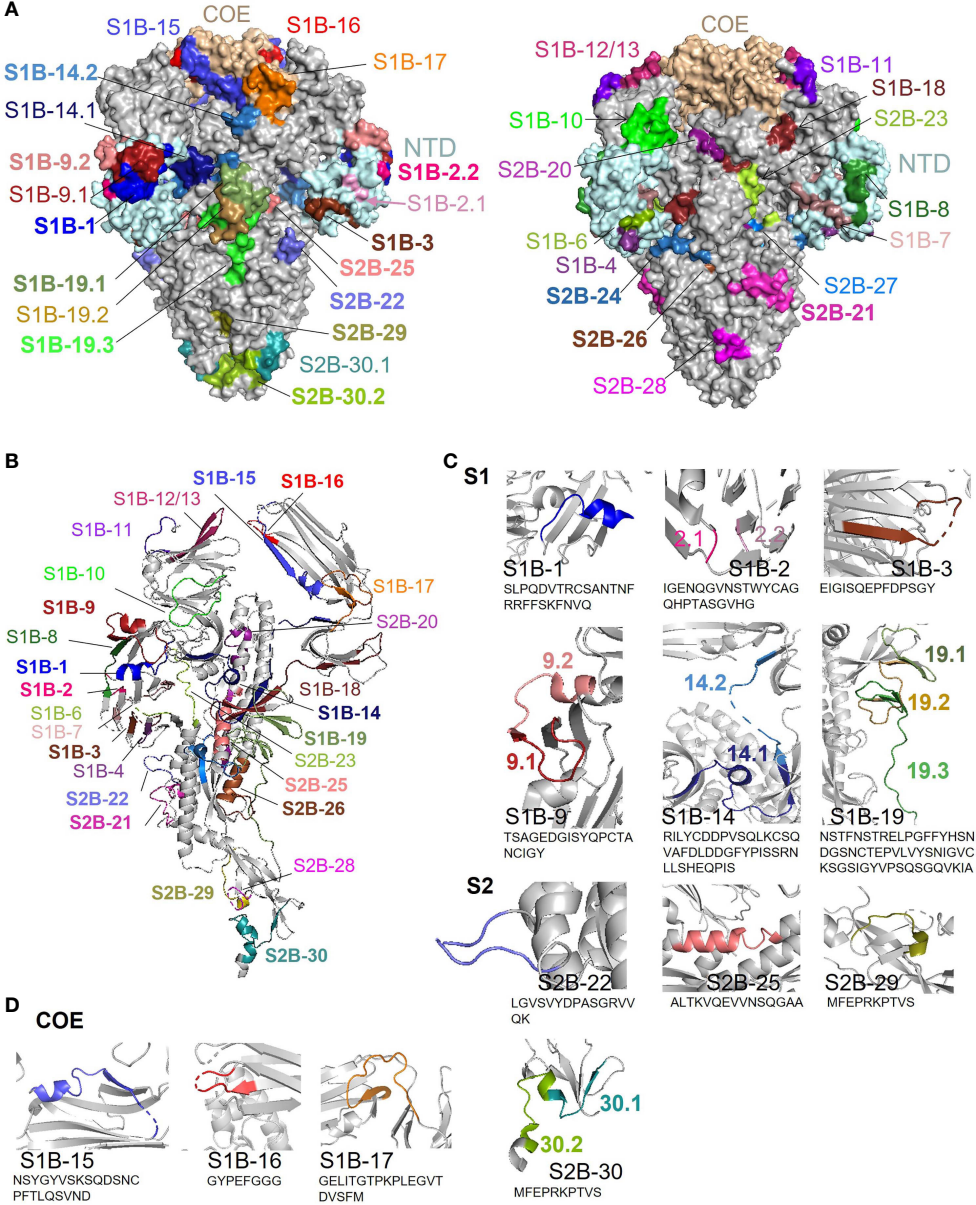
Surface representation and locations of the B-cell epitopes on the PEDV S protein. **(A)** Surface representation of the B-cell epitopes on the 3-D structure of the trimeric PEDV S protein (PDB: 6VV5) (35). The left panel demonstrates the epitopes in the COE and immunodominant epitopes; the right panel demonstrates all other epitopes with subdominant epitopes highlighted in bold. **(B)** Localization and secondary structure of the epitopes on the monomeric S protein. Immunodominant and subdominant epitopes are highlighted in bold. **(C)** Close-ups of immunodominant epitopes. **(D)** Close-ups of neutralizing epitopes in the COE domain.

Surface representation of all epitopes is depicted on the prefusion structure of the trimeric S protein of the PEDV strain USA/Colorado/2013 (PDB: 6VV5) (35) using PyMOL 2.3.4. As shown in **Figure 9A**, most of the epitopes are exposed on the surface, by which surface accessibility is one main feature associated with B-cell epitopes. Although coil is generally recognized as one main characteristic of the linear B-cell epitopes, B-cell epitopes identified in our study were found to be composed of various structures including coil, alpha helix, and beta sheet (**Figure 9B**). However, close-ups of the immunodominant epitopes and neutralizing epitopes in the COE revealed that these epitopes are either partly or entirely composed of coil structure and some epitopes also consist of other structures either alpha helix or beta sheet (**Figures 9C, D** **)**. Close-ups and the secondary structure of all the other epitopes are shown in **Supplementary Figure 10**.

## Discussion

The genetic diversity of PEDV, particularly in the gene encoding S protein (6), has limited the protective potency of the PEDV vaccine. Identification of neutralizing B-cell epitopes that are conserved across all diverse PEDV strains will contribute to the development of a more effective universal vaccine to combat all diverse strains of PEDV. So far, eight B-cell epitopes have been identified from five regions of the PEDV S protein. With the aim to identify B-cell epitopes from diverse strains of PEDV, immunoinformatics approach allows us to primarily screen for candidate B-cell epitopes from a large number of sequences retrieved from the database. By using multiple prediction tools, none of these prediction tools gave the prediction result that covers all four known epitopes [peptide M (23), SS2 (22), SS6 (22), and 2C10 (25)] when they were used alone. Our work is in agreement with the previous study showing that prediction using two or more tools increased both accuracy and probability in defining a genuine B-cell epitope (36). Thus, we developed our own prediction methods combining multiple immunoinformatics tools to predict and identify peptides with high potential as B-cell epitopes. Our immunoinformatics methods allowed us to identify important B-cell epitopes from diverse sequences of PEDV. Moreover, we have employed the prediction methods developed in this study in identifying B-cell epitopes of severe acute respiratory syndrome coronavirus 2 (SARS-CoV-2) (37), and we have proven that our immunoinformatics-based method is potent and useful in facilitating the identification of immunodominant epitopes with less time and cost compared with conventional methods.

After prediction, the predicted B-cell epitopes of the PEDV S and M proteins were verified with PEDV-positive sera. Pig sera with PEDV-neutralizing activity were screened by neutralization assay with CV777 lineage virus (ACC_PEDV). Both PEDV genotypes (G1 and G2) were found and reported to be the cause of several PEDV outbreaks in Thailand in the past recent years (7, 38); therefore, the pig sera we collected from different farms may be varied in terms of PEDV strain specificity. However, by using our CV777 lineage PEDV, we were able to measure the neutralization potential of the pig serum samples. This neutralizing activity is possibly mediated by antibodies recognizing conserved epitopes on the PEDV S protein. ELISA test confirmed the reactivity of the predicted peptides with PEDV-positive sera. However, the patterns of antibody responses in two different pig strains, F1 and F3, were different with some peptides. This may be influenced by several factors such as pig genetic background, age, duration, and dose of infection. Moreover, it is also possible that the infection in each farm is caused by different PEDV strains as different genotypes and subgenotypes of PEDV were reported in different regions of Thailand (7, 38).

Statistical analysis of antibody responses in both F1 and F3 pig sera defined 11 immunodominant within the PEDV S proteins. Interestingly, although PEDV (alphacoronavirus) and SARS-CoV-2 (betacoronavirus) belong to different genus, the landscape of immunodominant epitopes on the S protein of these two coronaviruses remarkably resembles. The following epitopes including i) S1B-1, ii) S1B-19 (19.1 and 19.3), iii) S2B-22, and iv) S2B-29 and S2B-30.2 are located at the following regions: i) the N-terminus next to the signal sequence; ii) CTD, upstream region of the S1/S2 cleavage site; iii) FP region; and iv) upstream region of the HR2, respectively, which have also been indicated to be the main sites of immunodominant epitopes on the SARS-CoV-2 S protein recently reported by our group (37) and Li et al. (39). Moreover, the epitopes S1B-15, S1B-16, and S1B-17, which are located within the COE domain, also mirror the three most dominant epitopes within the SARS-CoV-2 S RBD identified in our previous work (37). Additionally, the C-terminal endodomain of the S protein is another main site accommodating an immunodominant B-cell epitope of SARS-CoV-2 (37, 39–41) as well as the neutralizing epitope 2C10 with the GPRLQPY motif (epitope S2B-33 in this study) of PEDV (25).

The neutralizing activity of the antibodies targeting these novel epitopes was addressed using neutralization–inhibition assay, by which the target antibodies are depleted/blocked with their cognate peptides. This technique has been previously used to identify neutralizing epitopes from SARS-CoV-2 (40) and coxsackievirus A16, a causative agent of HFMD (human, foot, hand and mouth disease) (42). However, we observed that the inhibitory effect of a peptide on different sera was different for some peptides. This may be because of the varied levels of antibodies against each epitope in different sera. Surprisingly, we did not see the inhibitory effect of three immunodominant epitopes, S1B-14.2, S1B-19.1, and S2B-22, on any sera. This result can be explained by two possibilities: i) these epitopes are indeed non-neutralizing epitopes recognized by non-neutralizing antibodies, and ii) the peptides cannot compete with the epitope present on the virus particle, which is perhaps more complete than the peptide in terms of amino acid composition and conformation, in binding to their cognate antibodies; thus, the neutralizing activity of the serum is maintained.

The neutralizing epitopes we identified are located at the regions that are functionally important during virus infection. Epitopes S1B-1 to S1B-9 are located at the NTD of the S1 subunit, the region that is involved in binding to sialoglycoconjugate receptor on the host cell (43, 44). The neutralizing epitope S1B-10 is located at the S1 region that plays an important role in PEDV attachment to the host cell using sugar-binding activity (45). Moreover, the neutralizing epitope S1B 14.1 overlaps with the C-terminal sequence of the conformational epitope (aa. 435–485) formerly identified in the PEDV PT strain (12), suggesting that the overlapping sequence (457-RILYCDDPVSQLKCSQVAFDL-477) may function as a core neutralizing epitope in that region. The S1D domain located in the S1 CTD, which also includes our neutralizing epitope S1B-19, is well documented as an immunodominant epitope/domain and a target of neutralizing antibodies (11, 22, 35). Within the COE domain, three neutralizing epitopes (S1B-15, S1B-16, and S1B-17) identified in our study may be the main targets for neutralizing antibody recognition in the neutralizing conformational epitope (aa. 575–639) previously identified by Chang et al. (12). Generally, a conformational epitope requires correct conformation to interact with its target antibody; however, we demonstrate here that short linear peptides within the long conformational epitope can also be recognized by antibodies and binding of these short linear peptides to the antibody is sufficient to interfere the neutralizing activity of the antibody. On the other hand, it is also possible that the linear epitopes we identified are functionally complete as an epitope. This hypothesis is supported by one of our studies showing that epitope S1B-17 is immunodominant and elicited neutralizing antibody in mice immunized with PEDV COE (unpublished work). Moreover, epitopes identified with continuous sequence and thought to be a linear epitope may actually function in the form of conformational epitope as shown by Chang et al. (12) and one of our immunodominant linear epitopes, S1B-9.2, by which only non-denatured peptide but not the denatured one could bind to the antibody (data not shown).

While most of the known neutralizing B-cell epitopes are located in the S1 subunit, information of B-cell epitopes in the S2 subunit is limited with only one neutralizing epitope identified. The epitope 2C10 located at the C-terminus of the S2 domain has been identified as a neutralizing epitope (24, 25). In this study, in addition to the 2C10 epitope, more neutralizing B-cell epitopes in the S2 subunit were identified. The neutralizing epitope S2B-20 is located next to the S1/S2 cleavage site; thus, antibody binding to this region may interfere S1/S2 cleavage, resulting in a loss of viral infectivity. The coronavirus S2 subunit consists of fusion peptide (FP) and heptad repeat regions (HR) that play important roles in cell membrane fusion (46). In a betacoronavirus, SARS-CoV-2, the epitopes located in the FP and HR2 regions have been identified as immunodominant and neutralizing epitope (47). In our study, the neutralizing epitopes S2B-21 and S2B-23 map in the FP, a region responsible for protease cleavage and membrane fusion (44); thus, antibody binding to this region could affect these processes during viral cell entry. The neutralizing epitopes S2B-24, 25, and 26 are located within the HR1 region, whereas neutralizing epitopes S2B-27, 28, 29, 30, and 31 are located in the upstream region of the HR2 and S2B-32 is located within the HR2. The HR1 and HR2 domains of coronaviruses play important roles in membrane fusion (44); thus, blocking these regions with antibodies may result in inhibition of the membrane fusion process.

In addition to the S protein, we showed that M protein is also a target for virus neutralization. Based on neutralization–inhibition assay, epitopes M-1, M-2, and M-6 are the most promising as evidenced by the loss of neutralizing ability of both tested sera in the presence of these three peptides. The neutralization potential of antibodies against coronavirus M protein has been previously reported in the study of transmissible gastroenteritis virus (TGEV), a PEDV closely related alphacoronavirus, that the antibodies against the TGEV membrane protein had neutralizing activity in the presence of complement (48). Together with the profile of B-cell epitope within the M protein, our study shows for the first time that antibodies targeting the M protein could mediate PEDV neutralization. This new finding suggests that the next-generation PEDV vaccine may need to include either epitopes or the entire M protein in the vaccine design. Epitopes in the M protein are generally more conserved than those in the S protein; they thus have a potential use in the development of a detection kit.

As our B-cell epitopes are predicted based on consensus sequence of each group of the S and M proteins, the epitope sequence may not match 100% with all PEDV strains/variants. However, some epitopes are highly conserved as evidenced by a high percentage of strain coverage among global PEDV strains. Epitopes in the M protein were found to be more conserved compared with those in the S protein. Within the S protein, epitopes in the S2 subunit are more conserved than those in the S1 subunit. These conserved epitopes may serve as candidates for the development of a multi-epitope universal vaccine.

Taken together, the method combining immunoinformatics with immunoassays enabled the identification of novel neutralizing linear B-cell epitopes on the PEDV S and M proteins. Even though these B-cell epitopes are derived from a consensus sequence, some are highly conserved among the global PEDV strains, which represent a promising vaccine target for the development of a universal epitope-based vaccine as well as for antibody detection. Importantly, the immunoinformatics method developed in this study can serve as a useful tool for the prediction of linear B-cell epitopes from proteins of interest.

## Materials and Methods

### Protein Sequence Retrieval and Phylogenetic Analysis

Amino acid sequences of the PEDV S and M proteins were retrieved from NCBI (https://www.ncbi.nlm.nih.gov/protein/). The retrieved protein sequences were analyzed individually based on a defined name and length using Sublime Text 3 program. Based on sequence similarity, the amino acid sequences of the S and M proteins were grouped using alignment and phylogenetic tree tools in the SeaView program (version 4). In the SeaView program, these amino acid sequences were aligned using MUSCLE and grouped using a tree based on distance analysis, NJ method, ignore gap, and bootstrap 1,000 replication. The consensus sequence of each group was generated using the MUSCLE method in Unipro UGENE program (version 1.24.2; September 1, 2016) (49, 50). These consensus sequences were then subjected to epitope prediction.

### B-Cell Epitope Prediction

Immunoinformatics tools were exploited to predict B-cell epitopes and properties of the amino acid residues. Firstly, linear B-cell epitope prediction was performed using BepiPred-2.0 (http://www.cbs.dtu.dk/services/BepiPred/), which predicts linear B-cell epitopes from a protein sequence, using a random forest algorithm trained on epitopes and non-epitope amino acids determined from crystal structures, followed by performing sequential prediction smoothing (51). In our study, the residues with the threshold score of epitope probability above 0.5 were considered as parts of a B-cell epitope. Putative B-cell epitopes were selected based on the region with at least six consecutive residues predicted to have epitope probability above 0.5. Notably, BepiPred-2.0 also predicts and provides accessibility and coil probability of each amino acid residue. In addition, other properties of the proteins were also characterized using multiple immunoinformatics tools provided by IEDB (http://tools.iedb.org/bcell/), except IUPred. IUPred (https://iupred.elte.hu/) was used to predict intrinsically unstructured proteins which infers to coil probability (52). The method of Emini (53) was used to predict surface accessibility, while the method of Kolaskar and Tongaonkar, the semi-empirical method (54), was used to predict antigenicity determinant. Hydrophilicity of the protein was predicted using the method of Parker. The results of prediction from each method were aligned and three methods for identifying and selecting candidate B-cell epitopes were created based on the four known B-cell epitopes derived from PEDV protein as described in the result part.

### Epitope Conservancy Analysis

As epitopes were predicted based on consensus sequences of each group of the PEDV S (14 groups) and M (5 groups) proteins, we further analyzed the conservancy of our predicted epitopes. Epitope conservancy was analyzed by calculating the percent strain coverage of the predicted epitopes in comparison to all of the sequences in the group using the formula below.


$$\%\text{ strain coverage}=\frac{\text{Number of accession sequences with }100\text{\% match to the predicted epitope}}{\text{Total accession number in the group}}\times 100$$

### Peptide Synthesis and Preparation

Peptides corresponding to the predicted epitopes were chemically synthesized (Mimotopes, Australia). All synthetic peptides include i) 57 predicted epitopes; ii) 3 known reference B-cell epitopes, SS2 (YSNIGVCK) (22), SS6 (LQDGQVKI) (22), and 2C10 (GPRLQPY) (25); and iii) irrelevant peptides, N(MHC) (LADSYEITY) and E(CTL) (AVYTPIGRLY). Synthetic peptides were dissolved to the concentration of 3 nmol/μl (3 mM) as a peptide stock in sterile distilled water containing 0.1% acetic acid. Peptide stocks were aliquoted and stored at −20°C until used.

### Animal Ethics and Preparation of Pig Sera

All procedures of sample collection from pigs raised in the farms were performed in accordance with the guidelines of the Institutional Animal Care and Use Committee (IACUC) of Institute for Animals for Scientific Purpose Development and carried out under the regulation and permission of King Mongkut’s University of Technology Thonburi IACUC protocol no. KMUTT-IACUC-2019/018. For the control group, pigs were raised in the animal research facility of Mahidol University. All procedures were carried out under the regulation and permission of the Faculty of Veterinary Science, Mahidol University IACUC protocol No. MUVS-KA-2021-01-01.

Blood samples (10 ml) were collected from pigs naturally infected with PEDV. Infected pigs were observed and diagnosed by a veterinarian based on signs and symptoms of PED. Pigs showing PED signs or had been diagnosed with PED within the past 4 weeks were used as subjects. Bloods were collected from two different pig strains: F1 sow (Danish Landrace, age 35–40 weeks old) and F3 fattening pigs (Danish Landrace × Large white × Danish Duroc, age 7–12 weeks old). Blood was collected from 13 female F1 pigs and 14 either male or female pigs raised in three different farms. Control sera were prepared from 10 uninfected F3 pigs (age 7 weeks old) raised in the animal research facility of Mahidol University. Bloods were left to clot at room temperature for approximately 2 h, followed by centrifugation at 1,500×*g* for 10 min in a refrigerated centrifuge to separate the serum. Serum was aliquoted and separated to new tubes and stored at −20°C until used.

### Cell and PEDV Preparation

PEDV used in this study is a lineage of PEDV CV777, designated ACC_PEDV (classification is shown in **Figures 1B, D** **)**. Vero cells were cultured in complete DMEM medium supplemented with 10% fetal bovine serum (FBS, HyClone) and 1% penicillin/streptomycin (Pen/Strep, Gibco), and then incubated at 37°C with 5% CO_2_. The seed virus was sequentially propagated in Vero cell with PEDV medium [DMEM supplemented with 0.02% yeast extract (HiMedia), 10 μg/ml of trypsin (Sigma), and 0.3% tryptose phosphate broth] (tryptose, 20 g/L; dextrose, 2 g/L; sodium chloride, 5 g/L; disodium hydrogen phosphate, 2.5 g/L). To prepare the PEDV stock, Vero cells were cultured in 10 T-175 flasks. When the cell confluency was over 90%, the old medium was removed and the cells were infected with PEDV resuspended in 20 ml of PEDV medium. When the cytopathic effect (CPE) reached 70%, the medium was harvested and pooled. Sodium chloride (NaCl) was added to the medium to a final concentration of 0.5 M and polyethylene glycol (PEG, Sigma) was then added to a final concentration of 8%. After an overnight incubation, PEDV was harvested by centrifugation at 8,000 rpm for 20 min. The viral pellet was next resuspended in 5 ml of endotoxin-free PBS, aliquoted in a small volume to new tubes, and stored at −80°C. Virus titer was determined using 50% tissue culture infectious dose (TCID_50_) method. Virus suspension was diluted in 10-fold serial dilutions and pipetted into 10 wells of confluent Vero cells. After 3 days post-infection, PEDV-infected cells were observed and counted using immunofluorescence staining assay. TCID_50_ was next calculated.

### Immunofluorescence Staining Assay

Three days post-infection, Vero cells in 96-well plate were fixed with 1% formaldehyde at RT for 15 min and then washed with PBS once. Cells were permeabilized with cold 90% methanol and then incubated at 4°C for 5 min. Cells were washed once with PBS and blocked with 2% FBS/PBS for 1 h at RT. The blocking solution in each well was replaced with 50 µl of mouse anti-PEDV (Median Diagnostic) diluted 1:1,000 in PBS. Following an incubation for 2 h at RT, the cells were washed twice with PBS. Secondary antibody, donkey anti-mouse IgG conjugated with Alexa Fluor^®^ 594 (Abcam) diluted 1:1,000 in PBS, was added to the cell and incubated for 1 h at RT. The cells were washed twice with PBS and then the nucleus was stained with DAPI (Sigma) diluted 1:1,000 in PBS and incubated at RT for 30 min, followed by washing once with PBS. Fluorescence images were directly examined under an inverted fluorescence microscope (Olympus DP74).

### Antibody Neutralization Assays

All pig serum samples were 2-fold serially diluted with DMEM containing 1% Pen/Strep to the dilution ranging from 1:32 to 1:4,096. PEDV at the concentration of 200 TCID_50_/ml was prepared using PEDV medium. PEDV (10 TCID_50_ in 50 μl) was mixed with 50 μl of diluted serum or medium (control) and incubated at 37°C with 5% CO_2_. After, a 1-h incubation, the mixture of 100 μl was transferred into 96-well plates containing approximately 90% confluent monolayer of Vero cells. Each condition was performed in duplicate. After a 3-h incubation, the medium was removed and replaced with new PEDV medium. At day 3 post-infection, immunofluorescence staining assay was performed and the number of infected cells was counted. Endpoint neutralizing antibody titer was determined based on the serum dilution that completely inhibited virus infection. As all of the sera from the control pig did not neutralize PEDV at dilution 1:32 and higher, we thus set the cutoff of neutralizing antibody at neutralization titer 32 (reciprocal dilution titer). Only sera with neutralization titer ≥32 were subject to ELISA and neutralization and inhibition assay.

### ELISA

Synthetic peptides (0.75 nmol in 50 μl PBS/per well) were added into 96-well ELISA microplates (Greiner Bio-One). After an overnight incubation at 4°C, plates were washed three times using PBS containing 0.05% Tween 20 (PBST). Plates were blocked using 100 μl of PBST containing 5% FBS and incubated with agitation at room temperature (RT) for 1 h. Pig sera diluted 1:50 in PBST containing 1% FBS were added to the plates. After a 2-h incubation at RT, the plates were washed three times with PBST, and goat anti-pig IgG HRP antibody (Abcam) diluted 1:10,000 in PBST containing 1% FBS was added to the well (60 μl/well), followed by an incubation at RT for 90 min. After a three-time wash with PBST, the TMB substrate (BioLegend) was added (70 μl/well) and the plate was incubated in the dark at RT for 30 min, allowing the color to develop. The reaction was stopped by adding 35 μl of 1 M sulfuric acid (H_2_SO_4_), and the optical density was measured at a wavelength of 450 (OD450) (Multiskan FC, Thermo Scientific).

### Neutralization–Inhibition Assay

Neutralization–inhibition assay was conducted as previously described by Shi et al. (42) with some modifications. Sera from three F1 pigs (F1 1-3, F-1 1-4, and F1 1-5) and one F3 pig (F3 1-10) were subject to the assay. Serum was 2-fold serially diluted with DMEM containing 1% antibiotic to the dilution ranging from 1:32 to 1:256. PEDV was diluted in PEDV medium to 2,000 TCID_50_/ml. Synthetic peptides were mixed with the diluted serum to a final concentration of 200 nmol/ml in a volume of 50 μl. After an incubation at 37°C for 1 h, PEDV (100 TCID_50_ in 50 μl) was added to the peptide–serum mixture. After an incubation at 37°C for 1 h, the mixture (100 μl) was transferred to the Vero cells grown in 96-well plates. Following an incubation at 37°C with 5% CO_2_.for 3 h, the virus suspension was removed from the well and a new medium was added into the wells. In addition, the conditions that the Vero cells were incubated with i) medium alone, ii) serum alone, iii) PEDV alone, and iv) the mixture of PEDV and serum were also included in the assay as control conditions. All conditions were performed in duplicate. Following a 3-day (F1 1-4 and F1 1-5) or 5-day (F1 1-3 and F3 1-10) incubation, PEDV infection in Vero cell was investigated using immunofluorescence staining assay. Neutralization inhibition mediated by a peptide was determined at the endpoint neutralizing antibody titer.

### Labeling of Neutralizing B-Cell Epitopes

Putative neutralizing B-cell epitopes were aligned and compared with full-length S protein of PEDV USA/Colorado/2013 strain for the identification of epitope position. To localize each epitope within the trimeric PEDV S protein, the prefusion structure of the PEDV spike named 6VV5 (35) was used as a model. All predicted epitopes were depicted using PyMOL 2.3.4 program.

### Statistical Analysis

The statistical significance of the samples in different groups was analyzed using SPSS 22 for Windows software (SPSS, USA). Data analyses were performed using non-parametric Mann–Whitney *U* test. The difference between the two groups was determined based on *p <*0.05. Immunodominant and subdominant epitopes were defined with the following criteria. In response to a particular peptide, if all sera from both F1 and F3 pigs exhibited OD450 value higher than OD450 mean of the control group + 2SD, such peptide was considered an immunodominant. On the other hand, if all sera from both F1 and F3 pigs exhibited OD450 value higher than OD450 mean of the control group + 1SD, such peptide was considered a subdominant epitope.

## Data Availability Statement

The original contributions presented in the study are included in the article/**Supplementary Material**. Further inquiries can be directed to the corresponding author.

## Ethics Statement

The animal study was reviewed and approved by King Mongkut’s University of Technology Thonburi Institutional Animal Care and Use Committee (IACUC). Written informed consent for participation was not obtained from the owners because informed consent was obtained through verbal agreement between the veterinarian and farm owners.

## Author Contributions

KP designed and performed the experiments, analyzed and interpreted the data, wrote the original draft, and revised the manuscript. MR supervised the immunoinformatics methods and experimental design. PM provided the materials and reagent. KK prepared the uninfected pig sera. TH provided financial support, materials, and reagents. PJ collected the blood samples from PEDV-infected pigs. YR designed the experiments, analyzed and interpreted the data, provided supervision to KP, and reviewed and edited the manuscript. All authors read the manuscript and gave comments.

## Funding

This study received funding from B.F. Feed Company Limited. The funder was not involved in the study design, collection, analysis, interpretation of data, the writing of this article, or the decision to submit it for publication. The PhD study of KP is funded by the Development and Promotion of Science and Technology Talents Project (DPST).

## Conflict of Interest

Author TH is academic director of B.F. Feed Company Limited. PJ is employed by B.F. Feed Company Limited.

The remaining authors declare that the research was conducted in the absence of any commercial or financial relationships that could be construed as a potential conflict of interest.

## Publisher’s Note

All claims expressed in this article are solely those of the authors and do not necessarily represent those of their affiliated organizations, or those of the publisher, the editors and the reviewers. Any product that may be evaluated in this article, or claim that may be made by its manufacturer, is not guaranteed or endorsed by the publisher.
