# Supplementary material for: Immunodominant and Neutralizing Linear B-Cell Epitopes Spanning the Spike and Membrane Proteins of Porcine Epidemic Diarrhea Virus
Source: Front Immunol. 2022 Jan 19;12:785293. doi: 10.3389/fimmu.2021.785293 (PMC8807655; doi:10.3389/fimmu.2021.785293)
Supplement: Supplementary file 1 [file DataSheet_1.pdf]

frontiers

Supplementary Material

```
           ....|....| ....|....| ....|....| ....|....| ....|....| ....|....| ....|....| ....|....| ....|....| ....|....|
               5         15         25         35         45         55         65         75         85         95
M1         MSNGSIPVDE VIEHLRNWNF TW----NIIL TILLVVLQYG HYKYSAFLYG VKMAILWILW PLVLALSLFD AWASFQVNWV FFAFSILMAC ITLMLWIMYF
M2         MSNGSIPVDE VIQHLRNWNF TW----NIIL TILLVVLQYG HYKYSAFLYG VKMAILWILW PLVLALSLFD AWASFQVNWV FFAFSILMAC ITLMLWIMYF
M3         MSNGSIPVDE VIQHLRNWNF TW----NIIL TILLVVLQYG HYKYSAFLYG VKMAILWILW PLVLALSLFD AWASFQVNWV FFAFSILMAC ITLMLWIMYF
M4         MSNGSIPVDE VIQHLRNWNF TW----NIIL TILLVVLQYG HYKYSAFLYG VKMAILWILW PLVLALSLFD AWASFQVNWV FFAFSILMAC ITLMLWIMYF
M5         MSNGSIPVDE VIMLVLEHLR NWNFTWNIIL TILLVVLQYG HYKYSVFLYG VKMAILWILW PLVLALSLFD AWASFQVNWV FFAFSILMAC ITLMLWIMYF

           ....|....| ....|....| ....|....| ....|....| ....|....| ....|....| ....|....| ....|....| ....|....| ....|....|
              105        115        125        135        145        155        165        175        185        195
M1         VNSIRLWRRT HSWWSFNPET DALLTTSVMG RQVCIPVLGA PTGVTLTLLS GTLLVEGYKV ATGVQVSQLP NFVTVAKATT TIVYGRVGRS VNASSSTGWA
M2         VNSIRLWRRT HSWWSFNPET DALLTTSVMG RQVCIPVLGA PTGVTLTLLS GTLLVEGYKV ATGVQVSQLP NFVTVAKATT TIVYGRVGRS VNASSSTGWA
M3         VNSIRLWRRT HSWWSFNPET DALLTTSVMG RQVCIPVLGA PTGVTLTLLS GTLLVEGYKV ATGVQVSQLP NFVTVAKATT TIVYGRVGRS VNASSGTGWA
M4         VNSIRLWRRT HSWWSFNPET DALLTTSVMG RQVCIPVLGA PTGVTLTLLS GTLLVEGYKV ATGVQVSQLP NFVTVAKATT TIVYGRVGRS VNASSGTGWA
M5         VNSIRLWRRT HSWWSFNPET DALLTTSVMG RQVCIPVLGA PTGVTLTLLS GTLLVEGYKV ATGVQVSQLP NFVTVAKATT TIVYGRVGRS VNASSGTGWA

           ....|....| ....|....| ....|....|
              205        215        225
M1         FYVRSKHGDY SAVSNPSAVL TDSEKVLHLV
M2         FYVRSKHGDY SAVSNPSAVL TDSEKVLH--
M3         FYVRSKHGDY SAVSNPSAVL TDSEKVLHLV
M4         FYVRSKHGDY SAVSNPSSVL TDSEKVLHLV
M5         FYVRSKHGDY SAVSNPSAVL TDSEKVLHLV
```

Supplementary Figure 1. Alignment of consensus sequences of all 5 groups of the PEDV M protein.

```
         ....|....| ....|....| ....|....| ....|....| ....|....| ....|....| ....|....| ....|....| ....|....| ....|....|
             5         15         25         35         45         55         65         75         85         95
S1       MKSLTYFWLF LPVLSTLSLP QDVTRCSANT NFRRFFSKFN VQAPAVVVLG GY-LPIGENQ GVNSTWYCAG QHPTASGVHG IFVSHIRGGH GFEIGISQEP
S2       MKSLTYFWLF LPVLSTLSLP QDVTRCQSTI NFRRFFSKFN VQAPAVVVLG GY-LPSMNNQ GVSSSWYCGT GLETASGVHG IFLSYIDAGQ GFEIGISQEP
S3       MKSLTYFWLF LPVLSTLSLP QDVTRCSANT NFRRFFSKFN VQAPAVVVLG GY-LPIGENQ GVNSTWYCAG QHPTASGVHG IFVSHIRGGH GFEIGISQEP
S4       MKSLTYFWLF LPVLSTLSLP QDVTRCSANT NFRRFFSKFN VQAPAVVVLG GY-LPIGENQ GVNSTWYCAG QHPTASGVHG IFVSHIRGGH GFEIGISQEP
S5       MKSLTYFWLF LPVLSTLSLP QDVTRCSANT NFRRFFSKFN VQAPAVVVLG GY-LPIGENQ GVNSTWYCAG QHPTASGVHG IFLSHIRGGH GFEIGISQEP
S6       MKSLTYFWWF LPVLSTLSLP QDVTRCSANT NFRRFFSKFN VQAPAVVVLG GY-LPIGENQ GVNSTWYCAG QHPTASGVHG IFVSHIRGGH GFEIGISQEP
S7       MKSLTYFWLF LPVLSTLSLP QDVTRCSANT NFRRFFSKFN VQAPAVVVLG GY-LPIGENQ GVNSTWYCAG QHPTASGVHG IFVSHIRGGH GFEIGISQEP
S8       MRSLIYFWLL LPVLPTLSLP QDVSRCSANT NFRRFFSKFN VQAPAVVVLG GY-LPIGENQ GVNSTWYCAG QHPTASGVHG IFLSHIRGGH GFEIGISQEP
S9       MKSLTYFWLF LPVLSTLSLP QDVTRCSANT NFRRFFSKFN VQAPAVVVLG GY-LPIGENQ GVNSTWYCAG QHPTASGVHG IFLSHIRGGH GFEIGISQEP
S10      MKSLICFWLF LPVLSTFSLP QDVTRCAANT NFRRFFSKFN VQAPAVVVLG GY-LPIGENQ GVNSTWYCAG QHPTASGVHG IFLSHIRGGH GFEIGISQEP
S11      MTPLIYFWLF LPVLLTLSLP QDVTRCQSTI NFRRFFSKFN VQAPAVVVLG GY-LPSMNSS SWYCGTGIET DSGVHGIFLS YIDS----GQ GFEIGISQEP
S12      MRSLTYFWLF LPVLSTLSLP QDVTRCSANT NFRRFFSKFN VQAPAVVVLG GY-LPSGEKQ GVNSTWYCAG QHPTASGVHG IFLSHIRGGH GFEIGISQEP
S13      MRSLIYFLLF VPVLPTLSLP QDVHRCSANT NFRRFFSKFN VQAPAVVVLG GY-LPIGENQ GVNSTWYCAG QHPTASGVHG IFLSHIRGGH GFEIGISQEP
S14      MRSLIYFWLF LPVLPTLSLP QDVTRCQSTT NFRRFFSKFN VQAPAVVVLG GYLPSMNKQV DSSSSWYCGT GIETASGVHG IFLSYIDSGQ GFEIGISQEP

         ....|....| ....|....| ....|....| ....|....| ....|....| ....|....| ....|....| ....|....| ....|....| ....|....|
           105        115        125        135        145        155        165        175        185        195
S1       FDPSGYQLYL HKATN----G NTNATARLRI CQFPSIKTLG PTANNDVTTG RNCLFNKAIP AHMSEHS--V VGITWDNDRV TVFSDKIYYF YFKNDWSRVA
S2       FDPSGYQLYL HKATN----G NHNAIARLRI CQFPDNKTLG PTVNNDVTTG RNCLFNKAIP AYMQDGKNIV VGITWDNDRV TVFADKIYHF YLKNDWSRVA
S3       FDPSGYQLYL HKATN----G NTNATARLRI CQFPSIKTLG PTANNDVTTG RNCLFNKAIP AHMSEHS--V VGITWDNDRV TVFSDKIYYF YFKNDWSRVA
S4       FDPSGYQLYL HKATN----G NTNATARLRI CQFPSIKTLG PTANNDVTTG RNCLFNKAIP AHMSEHS--V VGITWDNDRV TVFSDKIYYF YFKNDWSRVA
S5       FDPSGYQLYL HKATN----G NTNATARLRI CQFPSIKTLG PTANNDVTTG RNCLFNKAIP AHMSEHS--V VGITWDNDRV TVFSDKIYYF YFKNDWSRVA
S6       FDPSGYQLYL HKATN----G NTNATARLRI CQFPSIKTLG PTANNDVTTG RNCLFNKAIP AHMSEHS--V VGITWDNDRV TVFSDKIYYF YFKNDWSRVA
S7       FDPSGYQLYL HKATN----G NTNATARLRI CQFPSIKTLG PTANNDVTTG RNCLFNKAIP AHMSEHS--V VGITWDNDRV TVFSDKIYYF YFKNDWSRVA
S8       FDPSGYQLYL HKATN----G NTNATARLRI CQFPSSKTLG PTANNDVTTG RNCLFNKAIP AHMSEHS--V VGITWDNDRV TVFSDKIYHF YFKNDWSRVA
S9       FDPSGYQLYL HKATN----G NTNATARLRI CQFPSIKTLG PTANNDVTTG RNCLFNKAIP AHMSEGKHSV VGITWDNDRV TVFSDKIYYF YFKNDWSRVA
S10      FDPSGYQLYL HKATN----G NTNATARLRI CQFPSIKTLT ATANDDVTTG RNCLFNKAIP AHMSEHS--V VGITWDNDRV TVFSDKIYHF YFKNDWSRVA
S11      FDPSGYQLYL HKATN----G NTSAIARLRI CQFPDNKTLG PT-VNDVTTG RNCLFNKAIP AHMSEHS--V VGITWDNDRV TVFSDKIYHF YFKNDWSRVA
S12      FDPSGYQLYL HKATN----G NTNATARLRI CQFPSNKTLG PTANNDVTTG RNCLFNKAIP AHMSEKHS-V VGITWDNDRV TVFADKIYHF YLKNDWSRVA
S13      FDPSGYQLYL HKATN----G NTNATARLRI CQFPSSKTLG PTANDDVTTG RNCLFNKAIP AHMSEHS--V VGITWDNDRV TVFSDKIYHF YFKNDWSRVA
S14      FDPSGYQLYL HKATNESQNG NTNAIARLRI CQFPDNKTLG PTVNDDVTTG RNCLFNKAIP AYMSDGKHIV VGITWDNDRV TVFADKIYHF YLKNDWSRVA

         ....|....| ....|....| ....|....| ....|....| ....|....| ....|....| ....|....| ....|....| ....|....| ....|....|
           205        215        225        235        245        255        265        275        285        295
S1       TKCYNSGGCA MQYVYEPTYY MLNVTSAGED GISYQPCTAN CIGYAANVFA TEPNGHIPEG FSFNNWFLLS NDSTLVHGKV VSNQPLLVNC LLAIPKIYGL
S2       TRCYNKRSCA MQYVYTPTYY MLNVTSAGED GIYYEPCTAN CSGYAANVFA TDSNGHIPEG FSFNNWFLLS NDSTLLHGKV VSNQPLLVNC LLAIPKIYGL
S3       TKCYNSGGCA MQYVYEPTYY MLNVTSAGED GISYQPCTAN CIGYAANVFA TEPNGHIPEG FSFNNWFLLS NDSTLVHGKV VSNQPLLVNC LLAIPKIYGL
S4       TKCYNSGGCA MQYVYEPTYY MLNVTSAGED GISYQLCTAN CIGYAANVFA TEPNGHIPEG FSFNNWFLLS NDSTLVHGKV VSNQPLLVNC LLAIPKIYGL
S5       TKCYNSGGCA MQYVYEPTYY TLNVTSAGED GISYQPCTAN CIGYAANVFA TEPNGHIPEG FSFNNWFLLS NDSTLVHGKV VSNQPLLVNC LLAIPKIYGL
S6       TKCYNSGGCA MQYVYEPTYY MLNVTSAGED GISYQPCTAN CIGYSANVFA TEPNGHIPEG FSFNNWFLLS NDSTLVHGKV VSNQPLLVNC LLAIPKIYGL
S7       TKCYNSGGCA MQYVYEPTYY MLNVTSAGED GISYQPCTAN CIGYAANVFA TEPNGHIPEG FSFNNWFLLS NDSTLVHGKV VSNQPLLVNC LLAIPKIYGL
S8       TKCYNSGGCA MQYVYEPTYY MLNVTSAGED GISYQPCTAN CIGYAANVFA TEPNGHIPEG FSFNNWFLLS NDSTLVHGKV VSNQPLLVNC LLAIPKIYGL
S9       TKCYNSGGCA MQYVYEPTYY MLNVTSAGED GISYQPCTAN CIGYAANVFA TEPNGHIPEG FSFNNWFLLS NDSTLVHGKV VSNQPLLVNC LLAIPKIYGL
S10      TKCYNSGGCA MQYVYEPIYY MLNVTSAGED GISYQPCTAN CIGYAANVFA TESNGHIPEG FSFNNWFLLS NDSTLFHGKV VSNQPLLVNC LLAIPKIYGL
S11      TKCYNSGGCA MQYVYEPTYY MLNVTSAGED GISYQPCTAN CIGYAANVFA TEPNGHIPEG FSFNNWFLLS NDSTLVHGKV VSNQPLLVNC LLAMPKIYGL
S12      TKCYNSGGCA MQYVYEPTYY MLNVTSAGED GISYQPCTAN CIGYAANVFA TEPNGHIPEG FSFNNWFLLS NDSTLLHGKV VSNQPLLVNC LLAIPKIYGL
S13      TKCYNSGGCA MQYVYEPTYY MLNVTSAGED GISYQPCTAN CIGYAANVFA TESNGHIPEG FSFNNWFLLS NDSTLVHGKV VSNQPLLVNC LLAIPKIYGL
S14      TRCYNSRSCA MQYVYTPTYY MLNVTSAGED GIYYEPCTAN CSGYAANVFA TDSNGHIPEG FSFNNWFLLS NDSTLLHGKV VSNQPLLVNC LWAIPKIYGL

         ....|....| ....|....| ....|....| ....|....| ....|....| ....|....| ....|....| ....|....| ....|....| ....|....|
           305        315        325        335        345        355        365        375        385        395
S1       GQFFSFNQTI DGVCNGAAVQ RAPEALRFNI NDTSVILAEG SIVLHTALGT NFSFVCSNSS NPHLATFAIP LGATQVPYYC FLKIVYGVVD TYNSTVYKFL
S2       GQFFSFNQTM DGVCNGAAAQ RAPEALRFNI NDTSVILAEG SIVLHTALGT NLSFVCSNSS DPHLATFAIP LGATQVPYYC FLK-----VD TYNSTVYKFL
S3       GQFFSFNQTI DGVCNGAAVQ RAPEALRFNI NDTSVILAEG SIVLHTALGT NFSFVCSNSS DPHLATFAIP LGAIQVPYYC FLK-----VD TYNSTVYKFL
S4       GQFFSFNQTI DGVCNGAAVQ RAPEALRFNI NDTSVILAEG SIVLHTALGT NFSFVCSNSS DPHLATFAIP LGATQVPYYC FLK-----VD TYNSTVYKFL
S5       GQFFSFNKTI DGVCNGAAVQ RAPEALRFNI NDTSVILAEG SIVLHTALGT NFSFVCGNSS DPHLATFAIP LGATQVPYYC FLK-----VD TYNSTVYKFL
S6       GQFFSFNQTI DGVCNGAAVQ RAPEALRFNI NDTSVILAEG SIVLHTALGT NFSFVCSNSS DPHLATFAIP LGATQVPYYC FLK-----VD TYNSTVYKFL
S7       GQFFSFNQTI DGVCNGAAVQ RAPEALRFNI NDTSVILAEG SIVLHTALGT NFSFVCSNSS DPHLATFAIP LGAIQVPYYC FLK-----VD TYNSTVYKFL
S8       GQFFSFNQTI DGVCNGAAVQ RAPEALRFNI NDTSVILAEG SIVLHTALGT NFSFVCSNSS DPHLATFAIP LGATQVPYYC FLK-----VD TYNSPVYKFL
S9       GQFFSFNQTI DGVCNGAAVQ RAPEALRFNI NDTSVILAEG SIVLHTALGT NFSFVCSNSS DPHLATFAIP LGAIQVPYYC FLK-----VD TYNSTVYKFL
S10      GQFFSFNQTM DGVCNGAAAQ RAPEALRFNI NDTSVILAEG SIVLHTALGT NLSFVCSNSS NPDLATFAIP LGATQVPYYC FLK-----VD IYNSTVYKFL
S11      GQFFSFNQTM DGVCNGAAVQ RAPEALRFNI NDTSVILAEG SIVLHTAFGT NLSFVCSNSS NPHLATFAIP LGATQVPYYC FLK-----VD TYNSTVYKFL
S12      GQFFSFNQTM DGVCNGAAAQ RAPEALRFNI NDTSVILAEG SIVLHTALGT NLSFVCSNSS DPHLATFAIP LGATQVPYYC FLK-----VD TYNSTVYKFL
S13      GQFFSFNQTI DGVCNGAAAQ RAPEALRFNI NDTSVILAEG SIVLHTALGT NFSFVCSNSS NPHLATFAIP LGATQVPYYC FLK-----VD TYNSTVYKFL
S14      GQFFSFNQTM DGVCNGAAAQ RAPEALRFNI NDTSVILAEG SIVLHTALGT NLSFVCSNSS DPHLAIFAIP LGATEVPYYC FLK-----VD TYNSTVYKFL

         ....|....| ....|....| ....|....| ....|....| ....|....| ....|....| ....|....| ....|....| ....|....| ....|....|
           405        415        425        435        445        455        465        475        485        495
S1       AVLPPTVREI VITKYGDVYV NGFGYLHLGL LDAVTINFTG HGTDDDVSGF WTIASTNFVD ALIEVQGTAI QRILYCDGDP VSQLKCSQVA FDLDDGFYPI
S2       AVLPPTVREI VITKYGDVYV NGFGYLHLGL LDAVTINFTG HGTDDDVSGF WTIASTNFVD ALIEVQGTAI QRILYCD-DP VSQLKCSQVA FDLDDGFYPI
S3       AVLPPTVREI VITKYGDVYV NGFGYLHLGL LDAVTINFTG HGTDDDVSGF WTIASTNFVD ALIEVQGTAI QRILYCD-DP VSQLKCSQVA FDLDDGFYPI
S4       AVLPPTVREI VITKYGDVYV NGFGYLHLGL LDAVTINFTG HGTDDDVSGF WTIASTNFVD ALIEVQGTAI QRILYCD-DP VSQLKCSQVA FDLDDGFYPI
S5       AVLPPTVREI VITKYGDVYV NGFGYLHLGL LDAVTINFTG HGTDDDVSGF WTIASTNFVD ALIEVQGTAI QRILYCD-DP VSQLKCSQVA FDLDDGFYPI
S6       AVLPPTVREI VITKYGDVYV NGFGYLHLGL LDAVTINFTG HGTDDDVSGF WTIASTNFVD ALIEVQGTAI QRILYCD-DP VSQLKCSQVA FDLDDGFYPI
S7       AVLPPTVREI VITKYGDVYV NGFGYLHLGL LDAVTINFTG HGTDDDVSGF WTIASTNFVD ALIEVQGTAI QRILYCD-DP VSQLKCSQVS FDLDDGFYPI
S8       AVLHPTVREI VITKYGDVYV NGFGYLHLGL LDAVTINFTG HGTDDDVSGF WTIASTNFVD ALIEVQGTAI QRILYCD-DP VSQLKCSQVA FDLDDGFYPI
S9       AVLPPTVREI VITKYGDVYV NGFGYLHLGL LDAVTINFTG HGTDDDVSGF WTIASTNFVD ALIEVQGTAI QRILYCD-DP VSQLKCSQVA FDLDDGFYPI
S10      AVLPSTVREI VITKYGDVYV NGFGYLHLGL LDAVTINFTG HGTDGDVSGF WTIASTNFVD ALIEVQGTAI QRILYCD-DP VSQLKCSQVA FDLDDGFYPI
S11      AVLPPTVREI VITKYGDVYV NGFGYLHLGL LDAVTINFTG HGTDDDVSGF WTIASTNFVD ALIEVQGTAI QRILYCD-DP VSQLKCSQVA FDLDDGFYPI
S12      AVLPPTVREI VITKYGDVYV NGFGYLHLGL LDAVTINFTG HGTDDDVSGF WTIASTNFVD ALIEVQGTAI QRILYCD-DP VSQLKCSQVA FDLDDGFYPI
S13      AVLPPTVREI VITKYGDVYV NGFGYLHLGL LDAVTINFTG HGTDDDVSGF WTIASTNFVD ALIEVQGTAI QRILYCD-DP VSQLKCSQVA FDLDDGFYPI
S14      AVLPPTVREI VITKYGDVYV NGFGYLHLGL LDAVTINFTG HGTDDDVSGF WTIASTNFVD ALIEVQGTAI QRILYCD-DP VSQLKCSQVA FDLDDGFYPI
```

```
           ....|....| ....|....| ....|....| ....|....| ....|....| ....|....| ....|....| ....|....| ....|....| ....|....|
                  505        515        525        535        545        555        565        575        585        595
S1         SSRNLLSHEQ PISFVTLPSF NDHSFVNITV SASFGGHSGA NLIASDTTIN GFSSFCVDTR QFTISLFYNV TNSYGYVSKS QDSNCPFTLQ SVNDYLSFSK
S2         SSRNLLSHEQ PISFVTLPSF NDHSFVNITV SASFGGHSGA NLIASDTTIN GFSSFCVDTR QFTISLFYNV TNSYGYVSKS QDSNCPFTLQ SVNDYLSFSK
S3         SSRNLLSHEQ PISFVTLPSF NDHSFVNITV SASFGGHSGA NLIASDTTIN GFSSFCVDTR QFTISLFYNV TNSYGYVSKS QDSNCPFTLQ SVNDYLSFSK
S4         SSRNLLSHEQ PISFVTLPSF NDHSFVNITV SASFGGHSGA NLIASDTTIN GFSSFCVDTR QFTISLFYNV TNSYGYVSNS QDSNCPFTLQ SVNDYLSFSK
S5         SSRNLLSHEQ PTSFVTLPSF NDHSFVNITV SASFGGLSGA NLIASDTTIN GFSSFCVDTR QFTISLFYNV TNSYGYVSKS QDSNCPFTLQ SVNDYLSFSK
S6         SSRNLLSHEQ PSSFVTLPSF NDHSFVNITV SASFGGHSGA NLIASDTTIN GFSSFCVDTR QFTISLFYNV TNSYGYVSNS QDSNCPFTLQ SVNDYLSFSK
S7         SSTNLLSHEQ PTSFVTLPSF NDHSFVNITV SAAFGGHSGA NLIASDTTIN GFSSFCVDTR QFTISLFYNV TNSYGYVSNS QDSNCPFTLQ SVNDYLSFSK
S8         SSRNLLSHEQ PISFVTLPSF NDHSFVNITV SASFGGHSGA NLIASDTTIN GFSSFCVDTR QFTISLFYNV TNSYGYVSKS QDSNCPFTLQ SVNDYLSFSK
S9         SSTNLLSHEQ PTSFVTLPSF NDHSFVNITV SAAFGGHSGA NLIASDTTIN GFSSFCVDTR QFTISLFYNV TNSYGYVSKS QDSNCPFTLQ SVNDYLSFSK
S10        SSRNLLSHEQ PTSFVTLPSF NDHSFVNIIV SAAFGGHSGA NLIASDTTIN GFSSFCVDTR QFTISLFYNV TNSYGYVSNS QDSNCPFTLQ SVNDYLSFSK
S11        SSRNLLSHEQ PISFVTLPSF NDHSFVNITV SASFGGPSGA NLIASDTTIN GFSSFCVDTR QFTISLFYNV TNSYGYVSKS QDSNCPFTLQ SVNDYLSFSK
S12        SSRNLLSHEQ PISFVTLPSF NDHSFVNITV SASFGGHSGA NLIASDTTIN GFSSFCVDTR QFTISLFYNV TNSYGYVSNS QDSNCPFTLQ SVNDYLSFSK
S13        SSRNLLSHEQ PTSFVTLPSF NDHSFVNITV SASFGGHSGA NLVASDTTIN GFSSFCVDTR QFTIRLFYNV TNSYGYVSKS QDSNCPFTLQ SVNDYLSFSK
S14        SSRNLLSHEQ PISFVTLPSF NDHSFVNITV SAAFGGHSGA NLIASDTTIN GFSSFCVDTR QFTITLFYNV TNSYGYVSKS QDSNCPFTLQ SVNDYLSFSK

           ....|....| ....|....| ....|....| ....|....| ....|....| ....|....| ....|....| ....|....| ....|....| ....|....|
                  605        615        625        635        645        655        665        675        685        695
S1         FCVSTSLLAS ACTIDLFGYP EFGSGVKFTS LYFQFTKGEL ITGTPKPLEG VTDVSFMTLD VCTKYTIYGF KGEGIITLTN SSFLAGVYYT SDSGQLLAFK
S2         FCVSTSLLAS ACTIDLFGYP EFGSGVKFTS LYFQFTKGEL ITGTPKPLEG VTDVSFMTLD VCTKYTIYGF KGEGIITLTN SSFLAGVYYT SDSGQLLAFK
S3         FCVSTSLLAS ACTIDLFGYP EFGSGVKFTS LYFQFTKGEL ITGTPKPLEG VTDVSFMTLD VCTKYTIYGF KGEGIITLTN SSFLAGVYYT SDSGQLLAFK
S4         FCVSTSLLAS ACTIDLFGYP EFGSGVKFTS LYFQFTKGEL ITGTPKPLEG VTDVSFMTLD VCTKYTIYGF KGEGIITLTN SSFLAGVYYT SDSGQLLAFK
S5         FCVSTSLLAS ACTIDLFGYP EFGSVVKFTS LYFQFTKGEL ITGTPKPLEG VTDVSFMTLD VCTKYTIYGF KGEGIITLTN SSFLAGVYYT SDSGQLLAFK
S6         FCVSTSLLAS ACTIDLFGYP EFGSGVKFTS LYFQFTKGEL ITGTPKPLEG VTDVSFMTLD VCTKYTIYGF KGEGIITLTN SSFLAGVYYT SDSGQLLAFK
S7         FCVSTSLLAS ACTIDLFGYP EFGSGVKFTS LYFQFTKGEL ITGTPKPLEG VTDVSFMTLD VCTKYTIYGF KGEGIITLTN SSFLAGVYYT SDSGQLLAFK
S8         FCVSTSLLAS ACTIDLFGYP DFGSGVKFTS LYFQFTKGEL ITGTPKPLEG VTDVSFMTLD VCTKYTIYGF KGEGIITLTN SSFLAGVYYT SDSGQLLAFK
S9         FCVSTSLLAS ACTIDLFGYP EFGSGVKFTS LYFQFTKGEL ITGTPKPLEG VTDVSFMTLD VCTKYTIYGF KGEGIITLTN SSFLAGVYYT SDSGQLLAFK
S10        FCVSTSLLAS ACTIDLFGYP EFGSGVKFTS LYFQFTKGEL ITGTPKPLEG VTDVSFMTLD VCTKYTIYGF KGEGIITLTN SSFLAGVYYT SDSGQLLAFK
S11        FCVSTSLLAS ACTIDLFGYP EFGGGVKFTS LYFQFTKGEL ITGTPKPLEG VTDVSFMTLD VCTKYTIYGF RGEGIITLTN SSFLAGVYYT SDSGQLLAFK
S12        FCVSTSLLAS ACTIDLFGYP DFGSGVKFTS LYFQFTKGEL ITGTPKPLEG VTDVSFMTLD VCTKYTIYGF KGEGIITLTN SSFLAGVYYT SDSGQLLAFK
S13        FCVSTSLLAS ACTIDLFGYP DFGSGVKFTS LYFQFTEGEL ITGTPKPLEG VTDVSFMTLD VCTKYTIYGF KGEGIITLTN SSFLAGVYYT SDSGQLLAFK
S14        FCVSTSLLAG ACTIDLFGYP AFGSGVKFTS LYFQFTKGEL ITGTPKPLEG VTDVSFMTLD VCTKYTIYGF KGEGIITLTN SSFLAGVYYT SDSGQLLAFK

           ....|....| ....|....| ....|....| ....|....| ....|....| ....|....| ....|....| ....|....| ....|....| ....|....|
                  705        715        725        735        745        755        765        775        785        795
S1         NVTSGAVYSV TPCSFSEQAA YVDDDIVGVI SSLSSSTFNS TRELPGFFYH SNDGSNCTEP VLVYSNIGVC KSGSIGYVPS QSGQVKIAPT VTGNISIPTN
S2         NVTSGAVYSV TPCSFSEQAA YVDDDIVGVI SSLSSSTFNS TRELPGFFYH SNDGSNCTEP VLVYSNIGVC KSGSIGYVPS QSGQVKIAPT VTGNISIPTN
S3         NVTSGAVYSV TPCSFSEQAA YVDDDIVGVI SSLSSSTFNS TRELPGFFYH SNDGSNCTEP VLVYSNIGVC KSGSIGYVPS QSGQVKIAPT VTGNISIPTN
S4         NVTSGAVYSV TPCSFSEQAA YVDDDIVGVI SSLSNSTFNS TRELPGFFYH SNDGSNCTEP VLVYSNIGVC KSGSIGYVPS QSGQVKIAPT VTGNISIPTN
S5         NVTSGAVYSV TPCSFSEQAA YVDDDIVGVI SSLSNSTFNS TRELPGFFYH SNDGSNCTEP VLVYSNIGVC KSGSIGYVPS QSGQVKIAPT VTGNISIPTN
S6         NVTSGAVYSV TPCSFSEQAA YVDDDIVGVI SSLSNSTFNS TRELPGFFYH SNDGSNCTEP VLVYSNIGVC KSGSIGYVPS QSGQVKIAPT VTGNISIPTN
S7         NVTSGAVYSV TPCSFSEQAA YVDDDIVGVI SSLSSSTFNS TRELPGFFYH SNDGSNCTEP VLVYSNIGVC KSGSIGYVPS QSGQVKIAPT VTGNISIPTN
S8         NVTSGAVYSV TPCSFSEQAA YVDDDIVGVI SSLSSSTFNS TRELPGFFYH SNDGSNCTEP VLVYSNIGVC KSGSIGYVPL QGGQVKIAPT VTGNISIPAN
S9         NVTSGAVYSV TPCSFSEQAA YVDDDIVGVI SSLSSSTFNS TRELPGFFYH SNDGSNCTEP VLVYSNIGVC KSGSIGYVPS QSGQVKIAPT VTGNISIPTN
S10        NVTSGAVYSV TPCSFSEQAA YVDDDIVGVI SSLSNSTFNN TRELPGFFYH SNDGSNCTEP VLVYSNIGVC KSGSIGYVPS QSGQVKIAPT VTGNISIPTN
S11        NVTSGAVYSV TPCSFSEQAA YVDDDIVGVI SSLSNSTFNS TRELPGFFYH SNDGSNCTEP VLVYSNIGVC KSGSIGYVPS QSGQVKIAPT VTGNISIPTN
S12        NVTSGAVYSV TPCSFSEQAA YVDDDIVGVI SSLSNSTFNS TRELPGFFYH SNDGSNCTEP VLVYSNIGVC KSGSIGYVPS QSGQVKIAPT VTGNISIPTN
S13        NVTSGAVYSV TPCSFSEQAA YVDDDIVGVI SSLSNSTFNN TRELPGFFYH SNDGSNCTEP VLVYSNIGVC KSGSIGYVPS QSGQVKIAPT VTGNISIPTN
S14        NVTSGAVYSV TPCSFSEQAA YVDDDIVGVI SSLSNSTFNN TRELPGFFYH SNDGSNCTEP VLVYSNIGVC KSGSIGYVPL QDGQVKIAPT VTGNISIPTN

           ....|....| ....|....| ....|....| ....|....| ....|....| ....|....| ....|....| ....|....| ....|....| ....|....|
                  805        815        825        835        845        855        865        875        885        895
S1         FSMSIRTEYL QLYNTPVSVD CATYVCNGNS RCKQLLTQYT AACKTIESAL QLSARLESVE VNSMLTISEE ALQLATISSF NGDGYNFTNV LGVSVYDPAS
S2         FSMSIRTEYL QLYNTPVSVD CATYVCNGNS RCKQLLTQYT AACKTIESAL QLSARLESVE VNSMLTISEE ALQLATISSF NGDGYNFTNV LGVSVYDPAS
S3         FSMSIRTEYL QLYNTPVSVD CATYVCNGNS RCKQLLTQYT AACKTIESAL QLSARLESVE VNSMLTISEE ALQLATISSF NGDGYNFTNV LGVSVYDPAS
S4         FSMSIRTEYL QLYNTPVSVD CATYVCNGNS RCKQLLTQYT AACKTIESAL QLSARLESVE VNSMLTISEE ALQLATISSF NGDGYNFTNV LGVSVYDPAS
S5         FSMSIRTEYL QLYNTPVSVD CATYVCNGNS RCKQLLTQYT AACKTIESAL QLSARLESVE VNSMLTISEE ALQLATISSF NGDGYNFTNV LGVSVYDPAS
S6         FSMSIRTEYL QLYNTPVSVD CATYVCNGNS RCKQLLTQYT AACKTIESAL QLSARLESVE VNSMLTISEE ALQLATISSF NGDGYNFTNV LGVSVYDPAS
S7         FSMSIRTEYL QLYNTPVSVD CATYVCNGNS RCKQLLTQYT AACKTIESAL QLSARLESAE VNSMLTISEE ALQLATISSF NGDGYNFTNV LGVSVRDPAS
S8         FSMSIRTEYL QLYNTPVSVD CATYVCNGNS RCKQLLTQYT AACKTIESAL QLSARLESVE VNSMLTISEE ALQLATISSF NGDGYNFTNV LGVSVYDPAS
S9         FSMSIRTEYL QLYNTPVSVD CATYVCNGNS RCKQLLTQYT AACKTIESAL QLSARLESAE VNSMLTISEE ALQLATISSF NGDGYNFTNV LGVSVYDPAS
S10        FSMSIRTEYL QLYNMPVSVD CATYVCNGNS RCKQLLTQYT AACKTIESAL QLSARLESVE VNSMLTLSEE ALQLATISSF NGDGYNFTNV LGVSMYDPAS
S11        FSMSIRTEYL QLYNTPVSVD CATYVCNGNS RCKQLLTQYT AACKTIESAL QLSARLESVE VNSMLTISEE ALQLATISSF NGDGYNFTNV LGVSVYDPAS
S12        FSMSIRTEYL QLYNTPVSVD CATYVCNGNS RCKQLLTQYT AACKTIESAL QLSARLESVE VNSMLTISEE ALQLATISSF NGDGYNFTNV LGVSVYEPAS
S13        FSMSIRTEYL QLYNTPVSVD CATYVCNGNS RCKQLLTQYT AACKTIESAL QLSARLESVE VNSMLTTSEE ALQLATISSF NGDGYNFTNV LGVSVYDPAS
S14        FSMSIRTEYL QLYNTPVSVD CATYVCNGNS RCKQLLTQYT AACKTIESAL QLSARLESVE VNSMLTISEE ALQLATISSF NGDGYNFTNV LGVSVYDPAS

           ....|....| ....|....| ....|....| ....|....| ....|....| ....|....| ....|....| ....|....| ....|....| ....|....|
                  905        915        925        935        945        955        965        975        985        995
S1         GRVVQKRSFI EDLLFNKVVT NGLGTVDEDY KRCSNGRSVA DLVCAQYYSG VMVLPGVVDA EKLHMYSASL IGGMVLGGFT SAAALPFSYA VQARLNYLAL
S2         GRVVQKRSFI EDLLFNKVVT NGLGTVDEDY KRCSNGRSVA DLVCAQYYSG VMVLPGVVDA EKLHMYSASL IGGMVLGGFT SAAALPFSYA VQARLNYLAL
S3         GRVVQKRSFI EDLLFNKVVT NGLGTVDEDY KRCSNGRSVA DLVCAQYYSG VMVLPGVVDA EKLHMYSASL IGGMVLGGFT SAAALPFSYA VQARLNYLAL
S4         GRVVQKRSFI EDLLFNKVVT NGLGTVDEDY KRCSNGRSVA DLVCAQYYSG VMVLPGVVDA EKLHMYSASL IGGMVLGGFT SAAALPFSYA VQARLNYLAL
S5         GRVVQKRSFI EDLLFNKVVT NGLGTVDEDY KRCSNGRSVA DLVCAQYYSG VMVLPGVVDA EKLHMYSASL IGGMVLGGFT SAAALPFSYA VQARLNYLAL
S6         GRVVQKRSFI EDLLFNKVVT NGLGTVDEDY KRCSNGRSVA DLVCAQYYSG VMVLPGVVDA EKLHMYSASL IGGMVLGGFT AAAALPFSYA VQARLNYLAL
S7         GRVVQKRSFI EDLLFNKVVT NGLGTVDEDY KRCSNGRSVA DLVCAQYYSG VMVLPGVVDA EKLHMYSASL IGGMVLGGFT AAAALPFSYA VQARLNYLAL
S8         GRVVQKRSFI EDLLFNKVVT NGLGTVDEDY KRCSNGRAVA DLVCAQYYSG VMVLPGVVDA EKLHMYSASL IGGMVFGGFT AAAALPFSYA VQARLNYLAL
S9         GRVVQKRSFI EDLLFNKVVT NGLGTVDEDY KRCSNGRSVA DLVCAQYYSG VMVLPGVVDA EKLHMYSASL IGGMVLGGFT AAAALPFSYA VQARLNYLAL
S10        GRVVQKRSFI EDLLFNKVVT NGLGTVDEDY KRCSNGRSVA DLVCAQYYSG VMVLPGVVDA EKLHMYSASL IGGMVLGGFT AAAALPFSYA VQARLNYLAL
S11        GRVVQKGSFI EDLLFNKVVT NGLSTVDEDY KRCSNGRSVA DLVCAQYYSG VMVLPGVVDA EKLHMYSASL IGGMALGGLT TAAALPFSHA VQARLNYLAL
S12        GRVVHKRSFI EDLLFNKVVT NGLGTVDEDY KRCSNGRSVA DLVCAQYYSG VMVLPGVVDA EKLHMYSASL IGGMALGGFT AAAALPFSYA VQARLNYLAL
S13        GRVVHKRSFI EDLLFNKVVT NGLGTVDEDY KRCSNGRSVA DLVCAQYYSG VMVLPGVVDA EKLHMYSASL IGGMVLGGFT AAAALPFSYA VQARLNYLAL
S14        GRVVQKRSFI EDLLFNKVVT NGLGTVDEDY KRCSNGRSVA DLVCAQYYSG VMVLPGVVDA EKLHMYSASL IGGMALGGFT AAAALPFSYA VQARLNYLAL
```

```
         ....|....| ....|....| ....|....| ....|....| ....|....| ....|....| ....|....| ....|....| ....|....| ....|....|
              1005       1015       1025       1035       1045       1055       1065       1075       1085       1095
S1       QTDVLQRNQQ LLAESFNSAI GNITSAFESV KEAISQTSKG LNTVAHALTK VQEVVNSQGA ALTQLTVQLQ HNFQAISSSI DDIYSRLDIL SADVQVDRLI
S2       QTDVLQRNQQ LLAESFNSAI GNITSAFESV KEAISQTSKG LNTVAHALTK VQEVVNSQGA ALTQLTVQLQ HNFQAISSSI DDIYSRLDIL SADVQVDRLI
S3       QTDVLQRNQQ LLAESFNSAI GNITSAFESV KEAISQTSKG LNTVAHALTK VQEVVNSQGA ALTQLTVQLQ HNFQAISSSI DDIYSRLDIL SADVQVDRLI
S4       QTDVLQRNQQ LLAESFNSAI GNITSAFESV KEAISQTSKG LNTVAHALTK VQEVVNSQGA ALTQLTVQLQ HNFQAISSSI DDIYSRLDIL SADVQVDRLI
S5       QTDVLQRNQQ LLAESFNSAI GNITSAFESV KEAISQTSKG LNTVAHALTK VQEVVNSQGA ALTQLTVQLQ HNFQAISSSI DDIYSRLDIL TADVQVDRLI
S6       QTDVLQRNQQ LLAESFNSAI GNITSAFESV KEAISQTSKG LNTVAHALTK VQEVVNSQGA ALTQLTVQLQ HNFQAISSSI DDIYSRLDIL SADVQVDRLI
S7       QTDVLQRNQQ LLAESFNSAI GNITSAFESV KEAISQTSKG LNTVAHALTK VQEVVNSQGA ALTQLTVQLQ HNFQAISSSI DDIYSRLDIL SADVQVDRLI
S8       QTDVLQRNQQ LLAESFNSAI GNITSAFESV KEAISQTSKG LNTVAHALTK VQEVVNSQGA ALTQLTVQLQ HNFQAISSSI DDIYSRLDIL SADVQVDRLI
S9       QTDVLQRNQQ LLAESFNSAI GNITSAFESV KEAISQTSKG LNTVAHALTK VQEVVNSQGA ALTQLTVQLQ HNFQAISSSI DDIYSRLDIL SADVQVDRLI
S10      QTDVLQRNQQ LLAESFNSAI GNITSAFESV KEAVSQTSKG LNTVAHALTK VQEVVNSQGS ALTQLTVQLQ HNFQAISSSI DDIYSRLDIL SADVQVDRLI
S11      QTDVLQRNQQ LLAESFNSAI GNITSAFESV KEAISQTSNG LNTVAHALTK VQEVVNSQGS ALTQLTIQLQ HNFQAISSSI DDIYSRLDIL SADVQVDRLI
S12      QTDVLQRNQQ LLAESFNSAI GNITSAFESV KEAISQTSQG LNTVAHALTK VQEVVNSQGA ALSQLTIQLQ HNFQAISSSI DDIYSRLDIL SADVQVDRLI
S13      QTDVLQRNQQ LLAESFNSAI GNITSAFESV KEAISQTSQG LNTVAHALTK VQEVVNSQGA ALTQLTVQLQ HNFQAISSSI DDIYSRLDIL SADVQVDRLI
S14      QTDVLQRNQQ LLAESFNSAI GNITSAFESV KEAISQTSQG LNTVAHALTK VQEVVNSQGS ALTQLTIQLQ HNFQAISSSI DDIYSRLDIL SADVQVDRLI

         ....|....| ....|....| ....|....| ....|....| ....|....| ....|....| ....|....| ....|....| ....|....| ....|....|
              1105       1115       1125       1135       1145       1155       1165       1175       1185       1195
S1       TGRLSALNAF VAQTLTKYTE VQASRKLAQQ KVNECVKSQS QRYGFCGGDG EHIFSLVQAA PQG-----LL FLHTVLVPSD FVDVIAIAGL CVNDEIALTL
S2       TGRLSALNAF VAQTLTKYTE VQASRKLAQQ KVNECVKSQS QRYGFCGGDG EHIFSLVQAA PQG-----LL FLHTVLVPGD FVDVIAIAGL CVNDEIALTL
S3       TGRLSALNAF VAQTLTKYTE VQASRKLAQQ KVNECVKSQS QRYGFCGGDG EHIFSLVQAA PQG-----LL FLHTVLVPGD FVDVIAIAGL CVNDEIALTL
S4       TGRLSALNAF VAQTLTKYTE VQASRKLAQQ KVNECVKSQS QRYGFCGGDG EHIFSLVQAA PQG-----LL FLHTVLVPGD FVDVIAIAGL CVNDEIALTL
S5       TGRLSALNAF VAQTLTKYTE VQASRKLAQQ KVNECVKSQS QRYGFCGGDG EHIFSLVQAA PQG-----LL FLHTVLVPGD FVDVIAIAGL CVNDEIALTL
S6       TGRLSALNAF VAQTLTKYTE VQASRKLAQQ KVNECVKSQS QRYGFCGGDG EHIFSLWYRQ HVQAAPQGLL FLHTVLVPGD FVDVIAIAGL CVNDEIALTL
S7       TGRLSALNAF VAQTLTKYTE VQASRKLAQQ KVNECVKSQS QRYGFCGGDG EHIFSLVQAA PQG-----LL FLHTVLVPGD FVDVIAIAGL CVNDEIALTL
S8       TGRLSALNAF VAQTLTKYTE VQASRKLAQQ KVNECVKSQS QRYGFCGGDG EHIFSLVQAA PQG-----LL FLHTVLVPGD FVNVIAIAGL CVNDEIALTL
S9       TGRLSALNAF VAQTLTKYTE VQASRKLAQQ KVNECVKSQS QRYGFCGGDG EHIFSLVQAA PQG-----LL FLHTVLVPGD FVNVIAIAGL CVNDEIALTL
S10      TGRLSALNAF VAQTLTKYTE VQASRKLAQQ KVNECVKSQS QRYGFCGGDG EHIFSLVQAA PQG-----LL FLHTVLVPGD FVDVIAIAGL CVNDEIALTL
S11      TGRLSALNAF VAQTLTKYTE VQASRKLAQQ KVNECVKSQS QRYGFCGGDG EHIFSLVQAA PQG-----LL FLHTVLVPGD FVNVIAIDGL CINGDIALTL
S12      TGRLSALNAF VAQTLTKYTE VQASRRLAQQ KVNECVKSQS QRYGFCGGDG EHIFSLVQAA PQG-----LL FLHTVLVPGD FVNVTAIAGL CVNDDIALTL
S13      TGRLSALNAF VAQTLTKYTE VQASRKLAQQ KVNECVKSQS QRYGFCGGDG EHIFSLVQAA PQG-----LL FLHTVLVPGD FVNVIAIAGL CVNGDIALTL
S14      TGRLSALNAF VAQTLTKYTE VQASRKLAQQ KVNECVKSQS QRYGFCGGDG EHIFSLVQAA PQG-----LL FLHTVLVPGD FVNVIAIAGL CVNGDIALTL

         ....|....| ....|....| ....|....| ....|....| ....|....| ....|....| ....|....| ....|....| ....|....| ....|....|
              1205       1215       1225       1235       1245       1255       1265       1275       1285       1295
S1       REPGLVLFTH ELQNHTATEY FVSSRRMFEP RKPTVSDFVQ IESCVVTYVN LTRDQLPDVI PDYIDVNKTL DEILASLPNR TGPSLPLDVF NATYLNLTGE
S2       REPGLVLFTH ELQNHTATEY FVSSRRMFEP RKPTVSDFVQ IESCVVTYVN LTRDQLPDVI PDYIDVNKTL DEILASLPNR TGPSLPLDVF NATYLNLTGE
S3       REPGLVLFTH ELQNHTATEY FVSSRRMFEP RKPTVSDFVQ IESCVVTYVN LTRDQLPDVI PDYIDVNKTL DEILASLPNR TGPSLPLDVF NATYLNLTGE
S4       REPGLVLFTH ELQNHTATEY FVSSRRMFEP RKPTVSDFVQ IESCVVTYVN LTRDQLPDVI PDYIDVNKTL DEILASLPNR TGPSLPLDVF NATYLNLTGE
S5       REPGLVLFTH ELQNHTATEY FVSSRRMFEP RKPTVSDFVQ IQSCVVTYVN LTRDQLPDVI PDYIDVNKTL DEILASLPNR TGPSLPLDVF NATYLNLTGE
S6       REPGLVLFTH ELQNHTATEY FVSSRRMFEP RKPTVSDFVQ IESCVVTYVN LTRDQLPDVI PDYIDVNKTL DEILASLPNR TGPSLPLDVF NATYLNLTGE
S7       REPGLVLFTH ELQNHTATEY FVSSRRMFEP RKPTVSDFVQ IESCVVTYVN LTRDQLPDVI PDYIDVNKTL DEILASLPNR TGPSLPLDVF NATYLNLTGE
S8       REPGLVLFTH ELQNYNATEY FVSSRRMFEP RKPTASDFVQ IESCVVTYVN LTRDQLPDVI PDYIDVNKTL DEILASLPNR TGPSLPLDVF NATYLNLTGE
S9       REPGLVLFTH ELQ-DTATEY FVSSRRMYEP RKPTVGDFVQ IESCVVTYVN LTRDQLPEVI PDYIDVNKTL DEILASLPNR TGPSLSLDVF NATYLNLTGE
S10      REPGLVLFTH ELQNYTATEY FVSSRRMFEP RKPTVSDFVQ IESCVVTYVN LTRDQLPDVI PDYIDVNKTL DEILASLPNR TGPSLPLDVF NATYLNLTGE
S11      REPGLVLFTH ELQTYTATEY LVSSRRMFEP RKPTVSDFVQ IESCVVTYVN LTRDQLPDVI PDYIDVNKTL DEILASLPNR TGPSLPLDVF NATYLNLTGE
S12      REPGLVLFTH ELQTHTATEY FVSSRRMFEP RKPTVSDFVQ IESCVVTYVN LTSDQLPDVI PDYIDVNKTL DEILASLPNR TDPSFPLDVF NATYLNLTGE
S13      REPGLVLFTH ELQTHTATEY FVSSRRMFEP RKPTVSDFVQ IESCVVTYVN LTSDQLPDVI PDYIDVNKTL DEILASLPNR TGPSLPLDVF NATYLNLTGE
S14      REPGLVLFTH ELQTYTATEY FVSSRRMFEP RKPTVSDFVQ IESCVVTYVN LTSDQLPDVI PDYIDVNKTL DEILASLPNR TGPSLPLDVF NATYLNLTGE

         ....|....| ....|....| ....|....| ....|....| ....|....| ....|....| ....|....| ....|....| ....|....| ....|....|
              1305       1315       1325       1335       1345       1355       1365       1375       1385       1395
S1       IADLEQRSES LRNTTEELQS LIYNINNTLV DLEWLNRVET YIKWPWWVWL IIFIVLIFVV SLLVFCCIST GCCGCCGCCC ACFSGCCRGP RLQPYEVFEK
S2       IADLEQRSES LRNTTEELQS LIYNINNTLV DLEWLNRVET YIKWPWWVWL IIFIVLIFVV SLLVFCCIST GCCGCCGCCC ACFSGCCRGP RLQPYEVFEK
S3       IADLEQRSES LRNTTEELQS LIYNINNTLV DLEWLNRVET YIKWPWWVWL IIFIVLIFVV SLLVFCCIST GCCGCCGCCC ACFSGCCRGP RLQPYEVFEK
S4       IADLEQRSES LRNTTEELQS LIYNINNTLV DLEWLNRVET YIKWPWWVWL IIFIVLIFVV SLLVFCCIST GCCGCCGCCC ACFSGCCRGP RLQPYEVFEK
S5       IADLEQRSES LRNTTEELQS LIYNINNTLV DLEWLNRVET YIKWPWWVWL IIFIVLIFVV SLLVFCCIST GCCGCCGCCC ACFSGCCRGP RLQPYEVFEK
S6       IADLEQRSES LRNTTEELQS LIYNINNTLV DLEWLNRVET YIKWPWWVWL IIFIVLIFVV SLLVFCCIST GCCGCCGCCC ACFSGCCRGP RLQPYEVFEK
S7       IADLEQRSES LRNTTEELQS LIYNINNTLV DLEWLNRVET YIKWPWWVWL IIFIVLIFVV SLLVFCCIST GCCGCCGCCC ACFSGCCRGP RLQPYEVFEK
S8       IADLELRSES LRNTTEELQS LIYNINNTLV DLEWLNRVET YIKWPWWVWL IIFIVLIFVV SLLVFCCIST GCCGCCGCCC ACFSGCCRGP RLQPYEAFEK
S9       IADLEQRSES LRNTTEELQS LIYNINNTLV DLEWLNRVET YIKWPWWVWL IIFIVLIFVV SLLVFCCIST GCCGCCGCCG ACFSGCCRGP RLQPYEACTT
S10      IADLEQRSES LRNITVELQS LIYNINNTLV DFEWLNRVET YIKWPWWVWL IIFIVLIFVV SLLVFCCIST GCCGCCGCCG ACFSGCCRGP RFQPYEAFEK
S11      IADLEQRSES LRNTTEELRS LIYNINNTLV DLEWLNRVET YIKWPWWVWL IIFIVLIFVV SLLVFCCIST GCCGCCGCCG ACFSGCCRGP RLQPYEAFEK
S12      IADLEQRSES LRNTTEELRS LIYNINNTLV DLEWLNRVET YIKWPWWVWL IIFIVLIFVV SLLVFCCIST GCCGCCGCCC ACFSGCCRGP RLQPYEAFEK
S13      IADLEQRSES LQNITEELRS LIYNINNTLV DLEWLNRVET YIKWPWWVWL IIFIVLIFVV SLLVFCCIST GCCGCCGCCG ACFSGCCRGP RLQPYEAFEK
S14      IADLEQRSES LRNTTEELRS LIYNINNTLV DLEWLNRVET YIKWPWWVWL IIFIVLIFVV SLLVFCCIST GCCGCCGCCG ACFSGCCRGP RLQPYEAFEK

         ....|..
              1405
S1       VHVQ---
S2       VHVQ---
S3       VHVQ---
S4       VHVQ---
S5       VHVQ---
S6       VHVQ---
S7       VHVQ---
S8       VHVQ---
S9       FEKVHVQ
S10      VHVQ---
S11      VHVQ---
S12      VHVQ---
S13      VHVQ---
S14      VHVQ---
```

Supplementary Figure 2. Alignment of consensus sequences of all 14 groups of the PEDV S.

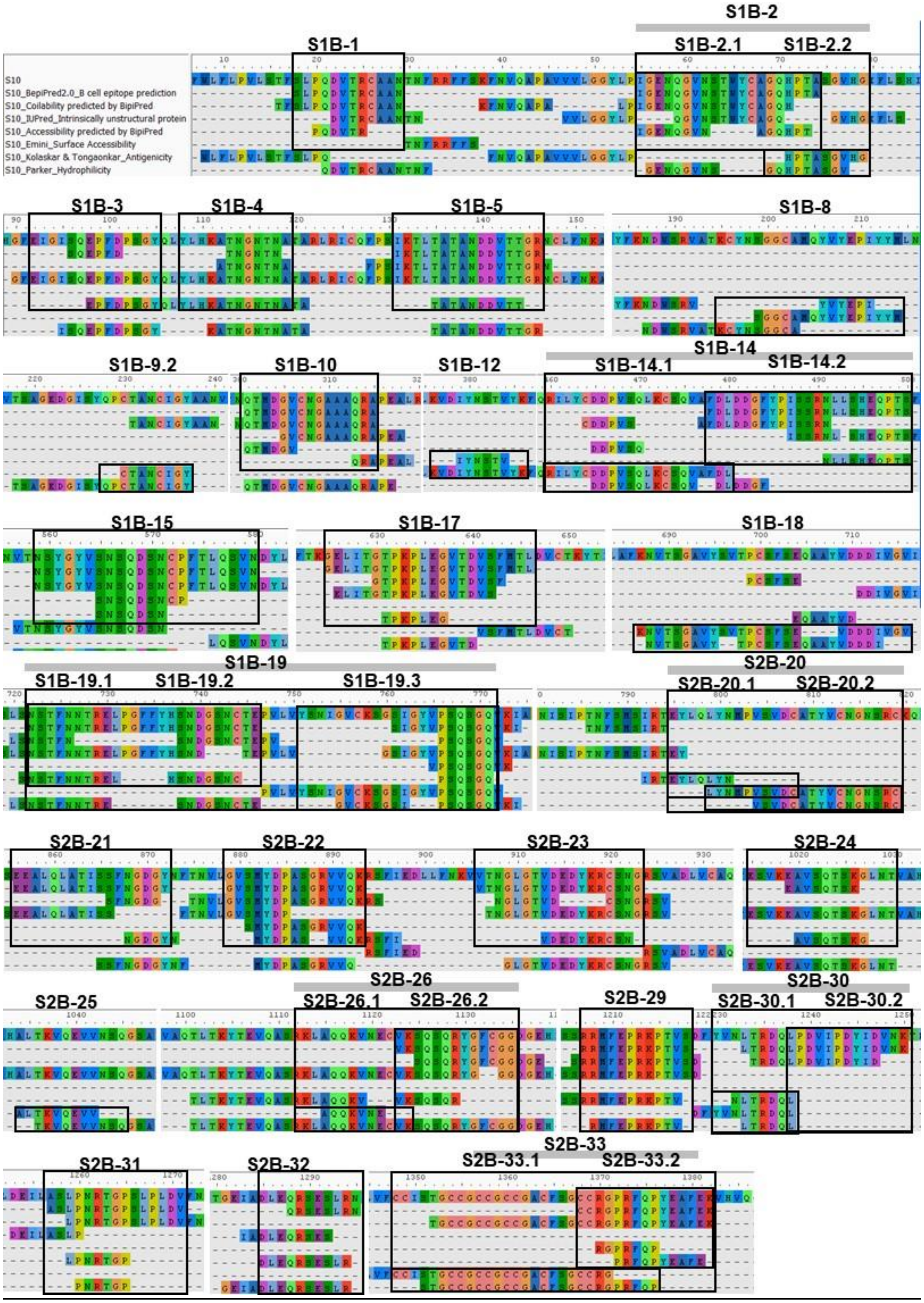

Supplementary Figure 3. B cell epitope prediction of the PEDV S protein. The consensus sequence of the S protein was subject to linear B cell epitope prediction using immunoinformatics methods A, B and C as described in Figure 2. Predicted epitopes are boxed and labeled with designated names. Prediction of the consensus sequence from group 10 is shown as a representative (out of 14 groups).

```
                           ....|....| ....|....| ....|....| ....|....| ....|....| ....|....| ....|....| ....|....| ....|....| ....|....|
                               5         15         25         35         45         55         65         75         85         95

M protein[CV777]           MSNGSIPVDE VIEHLRNWNF TWNIILTILL VVLQYGHYKY SVFLYGVKMA ILWILWPLVL ALSLFDAWAS FQVNWVFFAF SILMACITLM LWIMYFVNSI
M14[CH/SHH/06]             ---------- ---------- ---------- ---------- ---------- ---------- ---------- ---------- ---------- ----------
M epitopes in this study   ----SIPVDE VIQHLRNW-- ---------- VVLQYGHYKY SA-------- ---------- ---------- ---------- ---------- ----------

                           ....|....| ....|....| ....|....| ....|....| ....|....| ....|....| ....|....| ....|....| ....|....| ....|....|
                              105        115        125        135        145        155        165        175        185        195
M protein[CV777]           RLWRRTHSWW SFNPETDALL TTSVMGRQVC IPVLGAPTGV TLTLLSGTLL VEGYKVATGV QVSQLPNFVT VAKATTTIVY GRVGRSVNAS SGTGWAFYVR
M14[CH/SHH/06]             ---------- ---------- ---------- ---------- ---------- ---------- ---------- ---------- ---------- ----WAFYVR
M epitopes in this study   -----THSWW SFNPETD--- --SVMGRQVC IPVLGAPTGV T------TLL VEGYKVATGV QVSQLPNFV- ---------- GRVGRSVNAS SGTG------

                           ....|....| ....|....| ....|.
                              205        215        225
M protein[CV777]           SKHGDYSAVS NPSAVLTDSE KVLHLV
M14[CH/SHH/06]             ---------- ---------- ------
M epitopes in this study   SKHGDYSAVS NPSAVLTDSE KV----
```

Supplementary Figure 4. Alignment of the M protein of PEDV CV777 with the published epitopes and the epitopes identified in this study.

```
                ....|....| ....|....| ....|....| ....|....| ....|....| ....|....| ....|....| ....|....| ....|....| ....|....|
                    5         15         25         35         45         55         65         75         85         95
S protein       MRSLIYFWLL LPVLPTLSLP QDVTRCQSTT NFRRFFSKFN VQAPAVVVLG GYLPSMNSSS WYCGTGIETA SGVHGIFLSY IDSGQGFEIG ISQEPFDPSG
COE region      ---------- ---------- ---------- ---------- ---------- ---------- ---------- ---------- ---------- ----------
Known epitopes  ---------- ---------- ---------- ---------- ---------- ---------- ---------- ---------- ---------- ----------
Epitopes in     ---------- -------SLP QDVTRCSANT NFRRFFSKFN VQ-------- IGENQGVNST WYCAGQHPTA SGVHG----- -------EIG ISQEPFDPSG
this study

                ....|....| ....|....| ....|....| ....|....| ....|....| ....|....| ....|....| ....|....| ....|....| ....|....|
                   105        115        125        135        145        155        165        175        185        195
S protein       YQLYLHKATN GNTNAIARLR ICQFPDNKTL GPTVNDVTTG RNCLFNKAIP AYMRDGKDIV VGITWDNDRV TVFADKIYHF YLKNDWSRVA TRCYNRRSCA
COE region      ---------- ---------- ---------- ---------- ---------- ---------- ---------- ---------- ---------- ----------
Known epitopes  ---------- ---------- ---------- ---------- ---------- ---------- ---------- ---------- ---------- ----------
Epitopes in     Y--YLHKATN GNTNA----- ------IKTL GPTANNDVTT GR-------P AYMQDGKN-- ---------- -----KIYYF YFKNDWSRV- ----------
this study

                ....|....| ....|....| ....|....| ....|....| ....|....| ....|....| ....|....| ....|....| ....|....| ....|....|
                   205        215        225        235        245        255        265        275        285        295
S protein       MQYVYTPTYY MLNVTSAGED GIYYEPCTAN CTGYAANVFA TDSNGHIPEG FSFNNWFLLS NDSTLLHGKV VSNQPLLVNC LLAIPKIYGL GQFFSFNHTM
COE region      ---------- ---------- ---------- ---------- ---------- ---------- ---------- ---------- ---------- ----------
Known epitopes  MQYVYTPTYY ML-------- ---------- ---------- ---------- ---------- ---------- ---------- ---------- ----------
Epitopes in     --YVYEPTYY M---TSAGED GISYQPCTAN CIGY------ ---------- ---------- ---------- ---------- ---------- -------QTI
this study

                ....|....| ....|....| ....|....| ....|....| ....|....| ....|....| ....|....| ....|....| ....|....| ....|....|
                   305        315        325        335        345        355        365        375        385        395
S protein       DGVCNGAAVD RAPEALRFNI NDTSVILAEG SIVLHTALGT NLSFVCSNSS DPHLAIFAIP LGATEVPYYC FLKVDTYNST VYKFLAVLPP TVREIVITKY
COE region      ---------- ---------- ---------- ---------- ---------- ---------- ---------- ---------- ---------- ----------
Known epitopes  ---------- ---------- ---------- ---------- ---------- ---------- ---------- ---------- ---------- ----------
Epitopes in     DGVCNGAAAQ RA-------- ---------- ---------- -----CSNSS DPH------- --------C  FLKVDTYNST VYK------- ----------
this study                                                                          *KIV  YGVVDTYNST VYK

                ....|....| ....|....| ....|....| ....|....| ....|....| ....|....| ....|....| ....|....| ....|....| ....|....|
                   405        415        425        435        445        455        465        475        485        495
S protein       GDVYVNGFGY LHLGLLDAVT INFTGHGTDD DVSGFWTIAS TNFVDALIEV QGTSIQRILY CDDPVSQLKC SQVAFDLDDG FYPISSRNLL SHEQPISFVT
COE region      ---------- ---------- ---------- ---------- ---------- ---------- ---------- ---------- ---------- --------VT
Known epitopes  ---------- ---------- ---------- -VSGFWTIAS TNFVDALIEV QGTAIQRILY CDDPVSQLKC SQVAFDLDDG FY-------- ----------
Epitopes in     ---------- ---------- ---------- ---------- ---------- ------RILY CDDPVSQLKC SQVAFDLDDG FYPISSRNLL SHEQPIS---
this study

                ....|....| ....|....| ....|....| ....|....| ....|....| ....|....| ....|....| ....|....| ....|....| ....|....|
                   505        515        525        535        545        555        565        575        585        595
S protein       LPSFNDHSFV NITVSAAFGG LSSANLVASD TTINGFSSFC VDTRQFTITL FYNVTNSYGY VSKSQDSNCP FTLQSVNDYL SFSKFCVSTS LLAGACTIDL
COE region      LPSFNDHSFV NITVSAAFGG LSSANLVASD TTINGFSSFC VDTRQFTITL FYNVTNSYGY VSKSQDSNCP FTLQSVNDYL SFSKFCVSTS LLAGACTIDL
Known epitopes  ---------- ---------- ---------- ---------- ---------- ---------- ---------   TLQSVNDYL SFSKFCVSTS LLASACTIDL
                                                                                                      **TS LLASACTIDL
Epitopes in     ---------- ---------- ---------- ---------- ---------- -----NSYGY VSKSQDSNCP FTLQSVND-- ---------- ----------
this study

                ....|....| ....|....| ....|....| ....|....| ....|....| ....|....| ....|....| ....|....| ....|....| ....|....|
                   605        615        625        635        645        655        665        675        685        695
S protein       FGYPAFGSGV KLTSLYFQFT KGELITGTPK PLEGITDVSF MTLDVCTKYT IYGFKGEGII TLTNSSILAG VYYTSDSGQL LAFKNVTSGA VYSVTPCSFS
COE region      FGYPAFGSGV KLTSLYFQFT KGELITGTPK PLEGITDV-- ---------- ---------- ---------- ---------- ---------- ----------
Known epitopes  FGYPEFGSGV KFTSLYFQFT KGELITGTPK PLEGVT---- ---------- ---------- ---------- ---------- ---------- ----------
                FGYP
Epitopes in     -GYPEFGGG- ---------- -GELITGTPK PLEGVTDVSF M--------- ---------- ---------- ---------- ---KNVTSGA VYSVTPCSFS
this study

                ....|....| ....|....| ....|....| ....|....| ....|....| ....|....| ....|....| ....|....| ....|....| ....|....|
                   705        715        725        735        745        755        765        775        785        795
S protein       EQAAYVNDDI VGVISSLSNS TFNNTRELPG FFYHSNDGSN CTEPVLVYSN IGVCKSGSIG YVPSQYGQVK IAPTVTGNIS IPTNFSMSIR TEYLQLYNTP
COE region      ---------- ---------- ---------- ---------- ---------- ---------- ---------- ---------- ---------- ----------
Known epitopes  ---------- --------SS TFNSTREL-- ---------- ---PVLVYSN IGVCKSGSIG YVPLQDGQVK I--------- ---------- ----------
Epitopes in     EQAAYVDDDI VGV-----NS TFNSTRELPG FFYHSNDGSN CTEPVLVYSN IGVCKSGSIG YVPSQSGQVK IA-------- ---------- -EYLQLYNTP
this study

                ....|....| ....|....| ....|....| ....|....| ....|....| ....|....| ....|....| ....|....| ....|....| ....|....|
                   805        815        825        835        845        855        865        875        885        895
S protein       VSVDCATYVC NGNSRCKQLL TQYTAACKTI ESALQLSARL ESVEVNSMLT ISEEALQLAT ISSFNGDGYN FTNVLGASVY DPASGRVVQK RSVIEDLLFN
COE region      ---------- ---------- ---------- ---------- ---------- ---------- ---------- ---------- ---------- ----------
Known epitopes  ---------- ---------- ---------- ---------- ---------- ---------- ---------- ---------- ---------- ----------
Epitopes in     VSVDCATYVC NGNSRC---- ---------- ---------- ---------- --EEALQLAT ISSFNGDG-- ----LGVSVY DPASGRVVQK ----------
this study

                ....|....| ....|....| ....|....| ....|....| ....|....| ....|....| ....|....| ....|....| ....|....| ....|....|
                   905        915        925        935        945        955        965        975        985        995
S protein       KVVTNGLGTV DEDYKRCSNG RSVADLVCAQ YYSGVMVLPG VVDAEKLHMY SASLIGGMAL GGITAAAALP FSYAVQARLN YLALQTDVLQ RNQQLLAESF
COE region      ---------- ---------- ---------- ---------- ---------- ---------- ---------- ---------- ---------- ----------
Known epitopes  ---------- ---------- ---------- ---------- ---------- ---------- ---------- ---------- ---------- ----------
Epitopes in     --VTNGLGTV DEDYKRCSNG ---------- ---------- ---------- ---------- ---------- ---------- ---------- ----------
this study
```

```
                ....|....| ....|....| ....|....| ....|....| ....|....| ....|....| ....|....| ....|....| ....|....| ....|....|
                    1005       1015       1025       1035       1045       1055       1065       1075       1085       1095
S protein       NSAIGNITSA FESVKEAISQ TSKGLNTVAH ALTKVQEVVN SQGSALNQLT VQLQHNFQAI SSSIDDIYSR LDILSADVQV DRLITGRLSA LNAFVAQTLT
COE region      ---------- ---------- ---------- ---------- ---------- ---------- ---------- ---------- ---------- ----------
Known epitopes  ---------- ---------- ---------- ---------- ---------- ---------- ---------- ---------- ---------- ----------
Epitopes in     ---------- -ESVKEAISQ TSQG------ ALTKVQEVVN SQGAA----- ---------- ---------- ---------- ---------- ----------
this study

                ....|....| ....|....| ....|....| ....|....| ....|....| ....|....| ....|....| ....|....| ....|....| ....|....|
                    1105       1115       1125       1135       1145       1155       1165       1175       1185       1195
S protein       KYTEVQASRK LAQQKVNECV KSQSQRYGFC GGDGEHIFSL VQAAPQGLLF LHTVLVPGDF VNVLAIAGLC VNGEIALTLR EPGLVLFTHE LQTYTATEYF
COE region      ---------- ---------- ---------- ---------- ---------- ---------- ---------- ---------- ---------- ----------
Known epitopes  ---------- ---------- ---------- ---------- ---------- ---------- ---------- ---------- ---------- ----------
Epitopes in     --------RK LAQQKVNECV KSQSQRYGFC GG---WYRQH VQAAPQ---- ---------- ---------- ---------- -------THE LQNHTATEY-
this study
                ....|....| ....|....| ....|....| ....|....| ....|....| ....|....| ....|....| ....|....| ....|....| ....|....|
                    1205       1215       1225       1235       1245       1255       1265       1275       1285       1295
S protein       VSSRRMFEPR KPTVSDFVQI ESCVVTYVNL TSDQLPDVIP DYIDVNKTLD EILASLPNRT GPSLPLDVFN ATYLNLTGEI ADLEQRSESL RNTTEELRSL
COE region      ---------- ---------- ---------- ---------- ---------- ---------- ---------- ---------- ---------- ----------
Known epitopes  ---------- ---------- ---------- ---------- ---------- ---------- ---------- ---------- ---------- ----------
Epitopes in     -----MFEPR KPTVS----- ------YVNL TRDQLPDVIP DYIDVNK--- ---ASLPNRT GPSLPLDV-- ---------- ----QRSESL RNTTE-----
this study

                ....|....| ....|....| ....|....| ....|....| ....|....| ....|....| ....|....| ....|....| ...
                    1305       1315       1325       1335       1345       1355       1365       1375
S protein       INNINNTLVD LEWLNRVETY IKWPWWVWLI IVIVLIFVVS LLVFCCISTG CCGCCGCCGA CFSGCCRGPR LQPYEAFEKV HVQ
COE region      ---------- ---------- ---------- ---------- ---------- ---------- ---------- ---------- ---
Known epitopes  ---------- ---------- ---------- ---------- ---------- ---------- -------GPR LQPY------ ---
Epitopes in     ---------- ---------- ---------- ---------- ----CCISTG CCGCCGCCGA CFSGCCRGPR LQPYEAFEK- ---
this study
```

Supplementary Figure 5. Alignment of the PEDV CV777 S protein with the published epitopes and the epitopes identified in this study. * An epitope identified from consensus sequence of group 1; ** the known epitope, C2-1, which overlaps with epitope S1B-16.

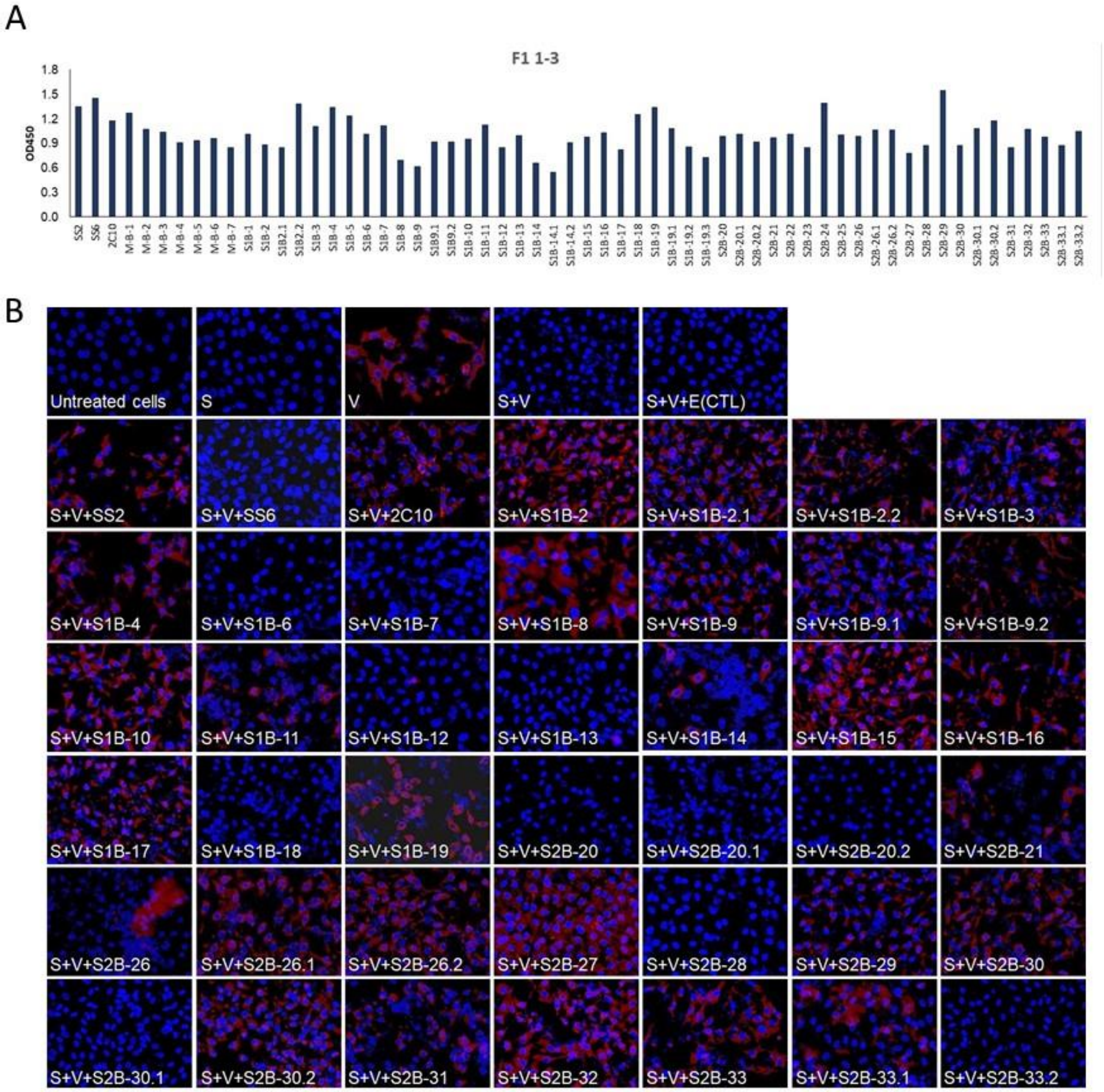

Supplementary Figure 6. Antibody response and neutralization-inhibition assay tested with F1 1-3 serum. (A) Antibody response against each peptide of the S and M protein in the F1 1-3 serum. (B) Neutralization-inhibition assay tested with F1 1-4 serum. Neutralization-inhibition assay was performed with the peptides indicated. The result was determined using immunofluorescence staining to detect infected cells. S: serum, V: virus. Peptides used in the assay are indicated in each condition.

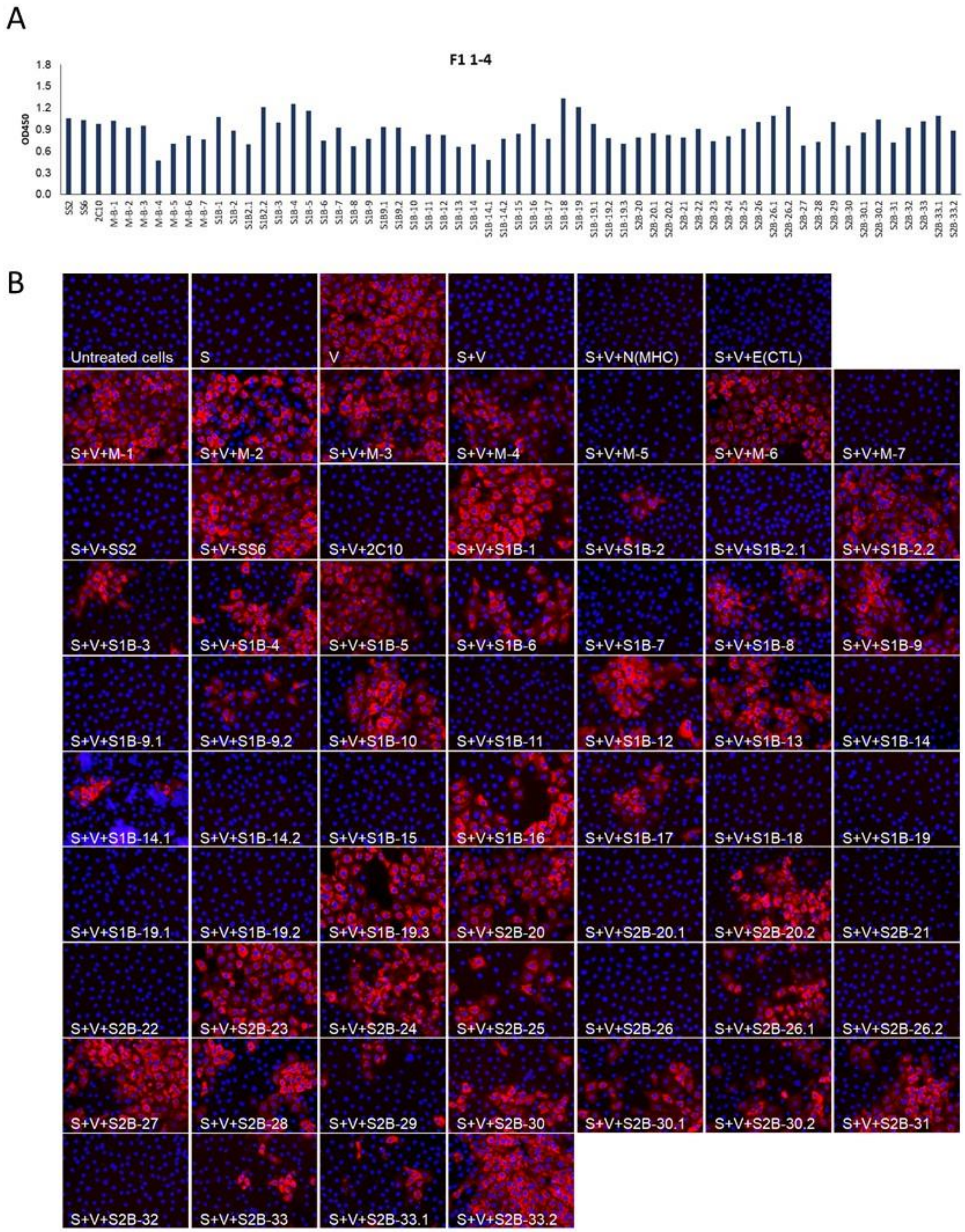

Supplementary Figure 7. Antibody response and neutralization-inhibition assay tested with F1 1-4 serum. (A) Antibody response against each peptide of the S and M protein in the F1 1-4 serum. (B) Neutralization-inhibition assay tested with F1 1-4 serum. Neutralization-inhibition assay was performed with the peptides indicated and the result was determined using immunofluorescence staining to detect infected cells. S: serum, V: virus. Peptides used in the assay are indicated in each condition.

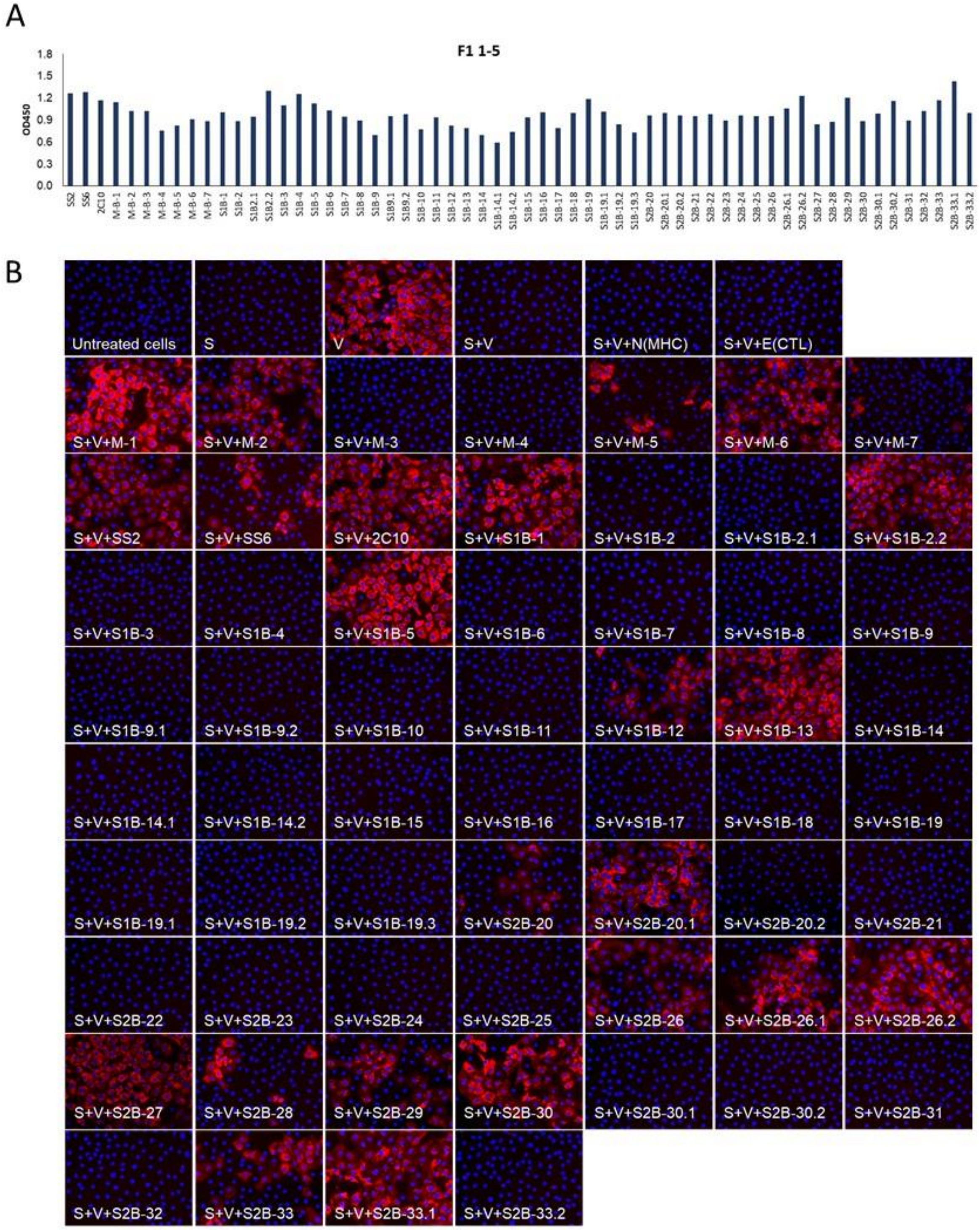

Supplementary Figure 8. Antibody response and neutralization-inhibition assay tested with F1 1-5 serum. (A) Antibody response against each peptide of the S and M protein in the F1 1-5 serum. (B) Neutralization-inhibition assay tested with F1 1-5 serum. Neutralization-inhibition assay was performed with the peptides indicated and the result was determined using immunofluorescence staining to detect infected cells. S: serum, V: virus. Peptides used in the assay are indicated in each condition.

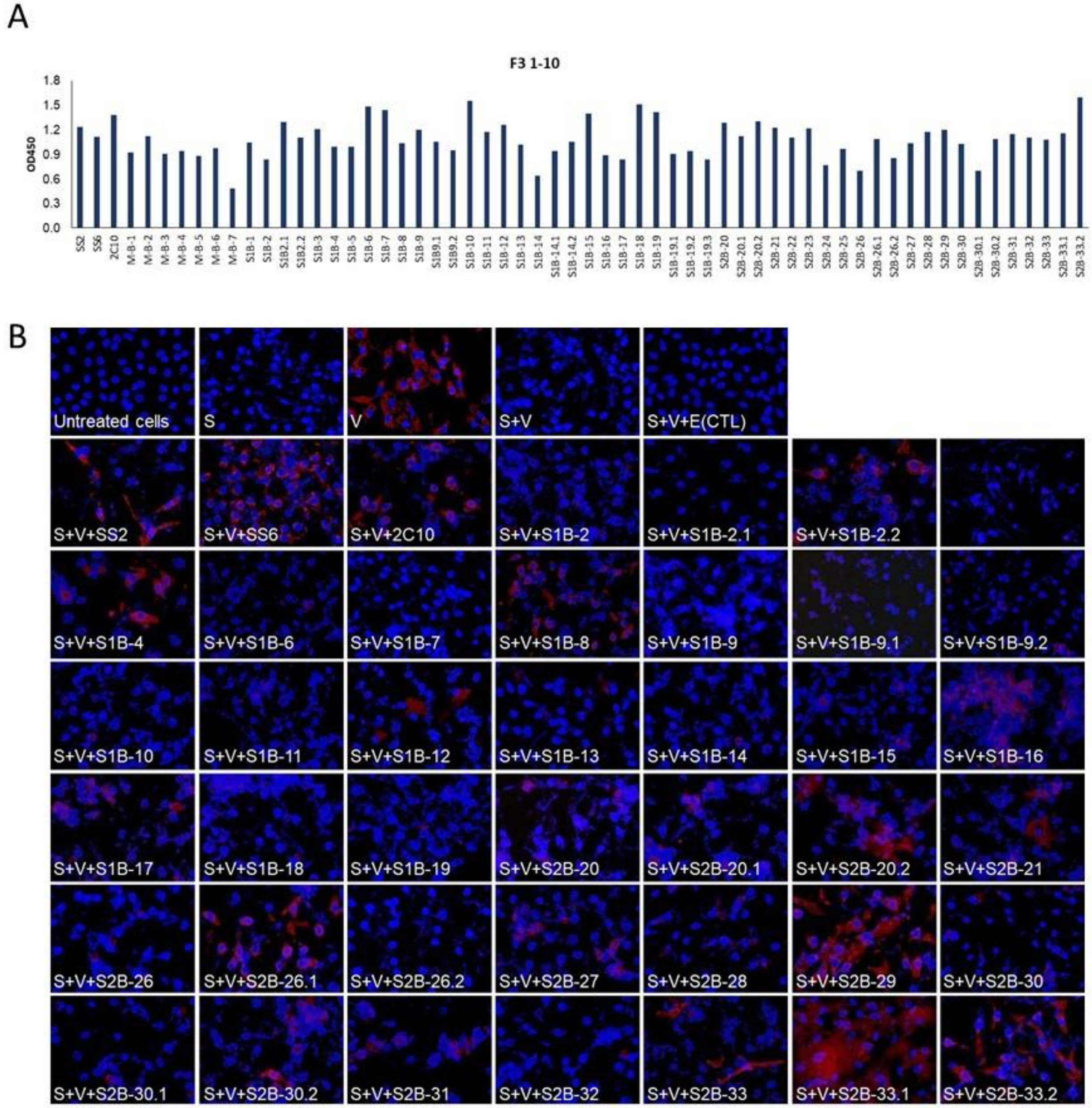

Supplementary Figure 9. Antibody response and neutralization-inhibition assay tested with F3 1-10 serum. (A) Antibody response against each peptide of the S and M protein in the F3 1-10 serum. (B) Neutralization-inhibition assay tested with F3 1-10 serum. Neutralization-inhibition assay was performed with the peptides indicated and the result was determined using immunofluorescence staining to detect infected cells. S: serum, V: virus. Peptides used in the assay are indicated in each condition.

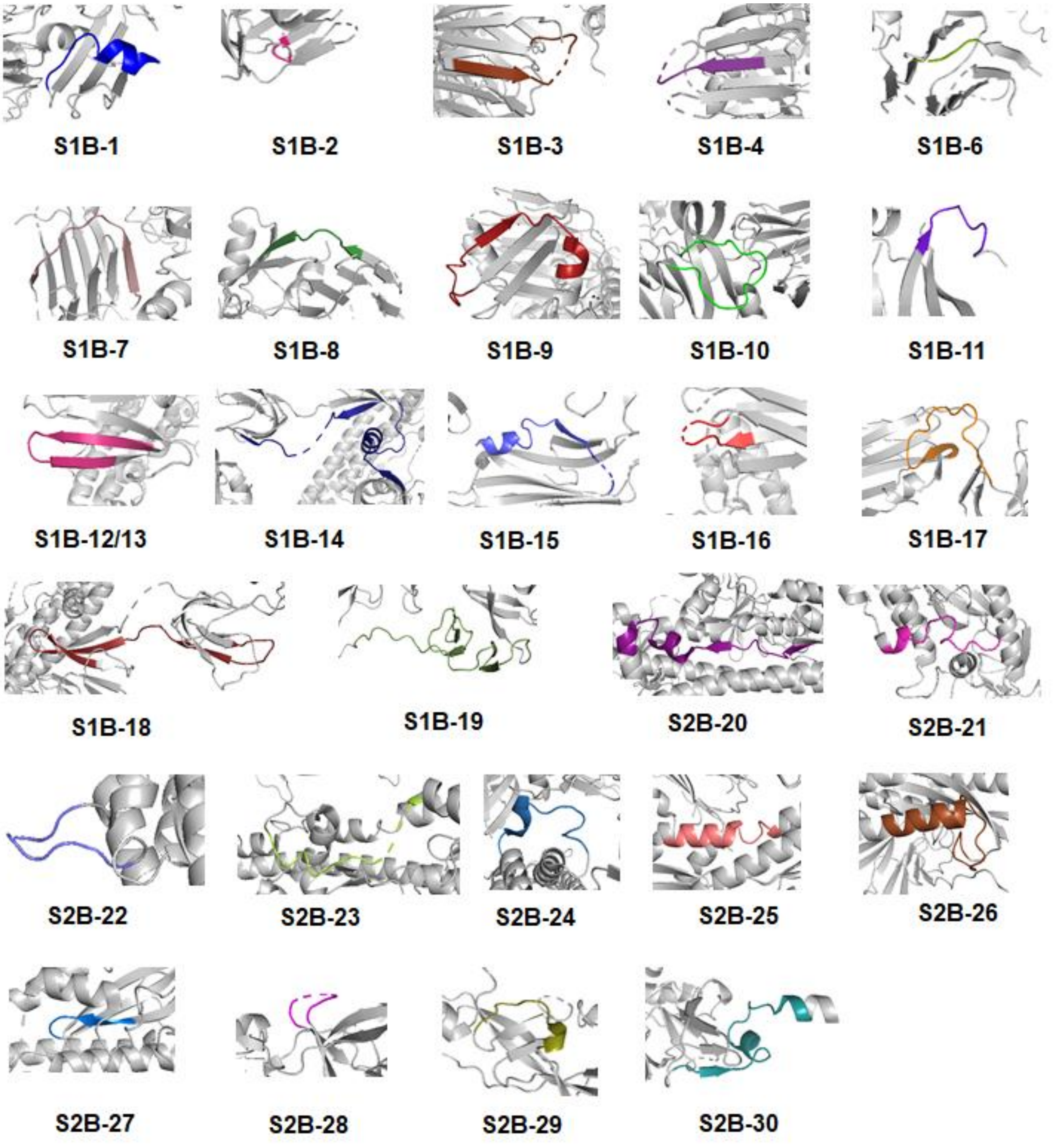

Supplementary Figure 10. Close-ups of all predicted epitopes. Predicted epitopes are labeled on the prefusion 3-D structure of monomeric PEDV S protein (6VV5 (1)). Note that epitope S1B-5 can not be labeled.

Supplementary Table 1. Endpoint neutralization titer of the pig serum samples.

Pig strain	Source	Pig (serum) name	Endpoint neutralization titer (reciprocal serum dilution)
F1 (Danish Landrace) female	Farm 1	F1 1-1	128
		F1 1-2	<32
		F1 1-3	512
		F1 1-4	128
		F1 1-5	128
		F1 1-6	64
		F1 1-7	64
		F1 1-8	32
		F1 1- 9	32
		F1 1-10	32
	Farm 2	F1 2-1	64
		F1 2-2	32
		F1 2-3	32
F3 (Danish Landrace x Large white x Danish Duroc) male and female	Farm 1	F3 1-1	32
		F3 1-2	64
		F3 1-3	32
		F3 1-4	32
		F3 1-5	32
		F3 1-6	<32
		F3 1-7	<32
		F3 1-8	32
		F3 1-9	<32
		F3 1-10	64
	Farm 3	F3 3-1	256
		F3 3-7	64
		F3 3-9	128
		F3 3-10	256
F3 male and femaile	Animal Research Facility	C-1	<32
		C-2	<32
		C-3	<32
		C-4	<32
		C-5	<32
		C-6	<32
		C-7	<32
		C-8	<32
		C-9	<32
		C-10	<32

1. Kirchdoerfer RN, Bhandari M, Martini O, Sewall LM, Bangaru S, Yoon K-J, et al. Structure and immune recognition of the porcine epidemic diarrhea virus spike protein. Structure. 2021;29(4):385-92.
